# The inclusion of habits in the stage model of self-regulated behavior change: an investigation of life events and red meat consumption in the UK

**DOI:** 10.3389/fpsyg.2024.1426171

**Published:** 2024-11-07

**Authors:** Colin Whittle, Nick Nash, Paul Haggar, Lorraine Whitmarsh

**Affiliations:** Department of Psychology, The University of Bath, Bath, United Kingdom

**Keywords:** stages of change, pro-environmental behavior, habitual behavior, meat consumption, climate change, habit discontinuity hypothesis

## Abstract

A shift to a diet with low or no red meat is considered necessary to end the environmental and health impacts caused by the current overconsumption of red meat. The self-regulated behavior change stage model (SSBC) proposes that people who intend to change their behavior progress through a series of discrete cognitive stages until, ultimately, they engage in the new behavior. However, what the consequences of habitual behaviors are for the initiation and progression through the stages of change have not yet been fully elucidated or investigated. We hypothesized that habitual behaviors that are antagonistic toward an alternative behavior will inhibit the initiation and progression through the stages of change. Furthermore, in line with the habit discontinuity hypothesis, we hypothesized that the experience of life events would counteract antagonistic habits and be positively associated with stages of change. Using a cross-sectional survey of people who consume red meat in the UK, our findings support the SSBC concept of stage-specific cognitive processes with goal intention and goal feasibility varying in importance depending on stage membership. However, personal norms were equally important for stage membership regardless of stage. Our hypotheses for antagonistic habits and life events were also partially supported; the antagonistic habit was not negatively associated with goal intention to change, but it was associated with a reduced likelihood of being in the final stage of change (i.e., of engaging in reduction). Experience of a life event was positively associated with goal intention to change, but it was negatively associated with being in a later stage of change. Overall, our findings provide novel theoretical insights into the role of habits and habit disruption in a stage model of behavior change. They also yield applied implications for understanding how to achieve a reduction in the over-consumption of red meat (or other, habitual, high greenhouse gas emitting behaviors) by supporting the importance of stage-tailored behavior change interventions and suggesting the potential to combine such stage-tailored intervention strategies with the strategy of targeting interventions to when existing habits are weakened due to context disruption.

## 1 Introduction

The overconsumption of red meat by people in a range of societies has had—and is continuing to have—catastrophic effects on the natural world and human health through its contribution to climate change, promotion of deforestation and resulting biodiversity loss, eutrophication, antibiotic resistance, and the development of non-communicable diseases, such as cardiovascular diseases and cancers (Rust et al., 2020; Willett et al., 2019). Although meat production is a complex system, with many different stakeholders to consider,^1^ a shift to a diet with low or no red meat is necessary to end these environmental impacts, improve health, and stay within safe planetary boundaries (Godfray et al., 2018; Willett et al., 2019). While many of the public in the United Kingdom (UK) support the need to reduce meat consumption for environmental, health, and ethical reasons (e.g., Climate Assembly, 2020), fewer have actually changed their behavior. Closing this attitude-intention-behavior gap (ElHaffar et al., 2020) requires an understanding of the drivers of behavior change.

Behavior change, including reducing red meat consumption, can be characterized as a conscious and deliberative process in which individuals progress through a series of discrete stages (Klöckner, 2014; Whitmarsh et al., 2024). Although a few stage models have been proposed (e.g., the transtheoretical model of behavior change; Prochaska, 2020), the stage model of self-regulated behavior change (SSBC; Bamberg, 2013b) was selected for investigation in the present study due to its theoretical focus on environmentally beneficial behavior changes (Bamberg, 2013b; Keller et al., 2019), such as red meat reduction (Klöckner, 2017). However, many human behaviors can become habitual, including dietary ones (Graves and Roelich, 2021) meaning that they are characterized as being unconscious and automatic (Gardner and Lally, 2023; Verplanken and Orbell, 2022). Given that progressing through the stages of behavior change is characterized as being conscious and deliberative (Bamberg, 2013a, 2020), we argue that existing habitual behavior may inhibit both the initiation of – and the progression through—the stages of behavior change. However, disruption to habits, such as from a life event, may enable conscious deliberation of alternative behaviors and so promote the initiation of—and the progression through—the stages of behavior change.

To our knowledge, habits have currently only been investigated once in relation to stage models of behavior change (Mei et al., 2024) and life events have yet to be investigated in relation to stage models of behavior change. As such, our research objective was to investigate the role of habits and life events in stage models of behavior change, questioning (1) do habits for current behaviors weaken intentions toward new behaviors? (2) do habits for current behaviors reduce the likelihood of progressing through stages of behavior change for a new behavior? (3) can experiencing a context disruption strengthen intentions toward new behaviors? and (4) can experiencing a context disruption increase the likelihood of progressing through stages behavior change? Through addressing these questions, we aim to elucidate on what the consequences of habitual behaviors are for the initiation and progression through the stages of change, which will inform intervention design and offer policy implications for behavior change.

In the following sections we will review the research literature that underpins our research questions and hypotheses, formalize the investigated hypotheses, describe the survey method, present the results of the survey, and discuss the research, the findings, and the implications we can draw.

## 2 Literature review

### 2.1 The stage model of self-regulated behavior change

Heckhausen and Gollwitzer (1987) proposed the Model of Action Phases (MAP), also known as the Rubicon Model of Action Phases (Keller et al., 2020), in which four discrete stages were described. Each stage is argued to have a cognitive “task” (Bamberg, 2013b, p. 152) which the individual is required to complete before moving to the next stage. The completion of each stage’s cognitive task is associated with the formation of an intention, which transitions them to the next stage (Gollwitzer, 1990). A brief description of each stage and the associated intention is given in Table 1.

**Table 1:** Summary descriptions of the behavior change stages and their associated intentions in the Model of Action Phases.

Stage name	Cognitive task	Intention formation
Pre-decisional	Deliberately reflect on one’s competing desires and translate some of those desires into a goal intention to engage in an alternative behavior	Goal intention, e.g., “I intend to eat less beef in the near future”^a^
Pre-actional	Deliberate on the pros and cons of different behavioral strategies for pursuing the held goal intention. Select one of those behavioral strategies and then form an intention to engage in it	Behavioral intention, e.g., “I intend to shift from eating beef meals to vegetarian meals”^a^
Actional	Identify specific opportunities to implement the chosen behavioral strategy. Begin engaging in the behavioral strategy	Implementation intention, e.g., “The next time I eat food, I have already planned to eat a vegetarian meal”^a^
Post-actional	Evaluate whether the goal has been achieved and if further action is required. Resisting any temptation to engage in the previous behavior.	

a Example intentions (in quotations) from Klöckner (2017, p. 445).

**Figure 1:**
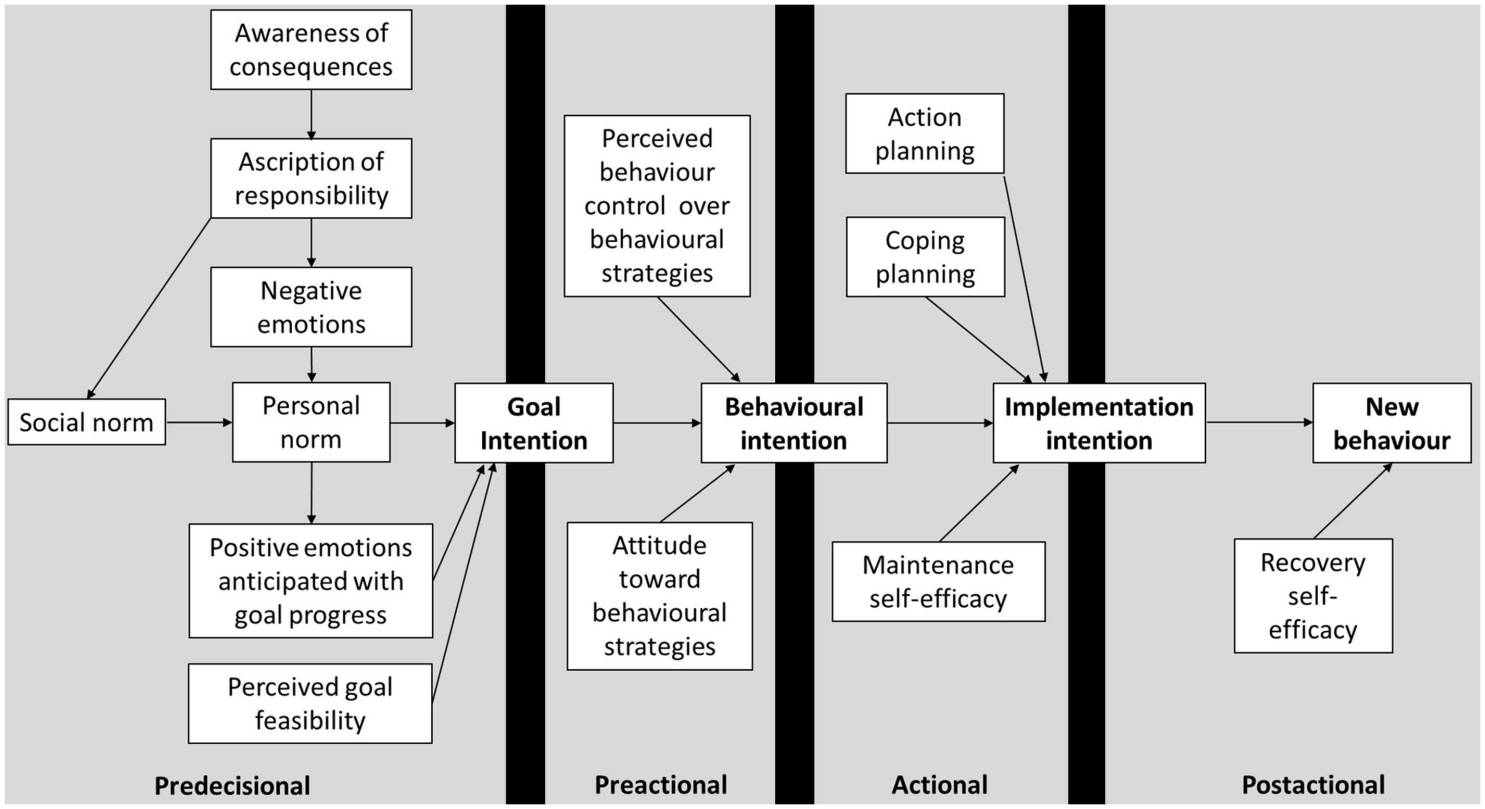
Conceptualization of the stage model of self-regulated behavior change (Bamberg, 2013b).

The original MAP has since been expanded by Bamberg (2013b) into the stage model of self-regulated behavior change (SSBC; Bamberg and Schulte, 2020). Critical to the development of the SSBC was Bamberg’s (2013b) recognition that, in the MAP’s Predecisional stage, existing behaviors are assumed to be performed as a habit, i.e., automatically and without conscious deliberation. However, the cognitive tasks and formation of intentions proposed in the MAP require conscious deliberation. To address this, Bamberg (2013b) drew on the cognitive factors proposed in the Norm Activation Model (NAM; Schwartz, 1977) and argued that a causal chain of awareness of consequences, ascription of responsibility, and negative emotions, such as guilt, will, in turn, cognitively activate an individual’s personal norms. Personal norms are feelings of obligation to act in line with held moral standards (Schwartz and Howard, 1984). As such, the activation of the individual’s personal norms might prompt them to deliberate on the inconsistency between their personal norms and their behavior, and consequently form a goal intention toward more consistent behavior (Bamberg, 2013b; Eriksson et al., 2008).^2^ The stages, their related intentions, and the supporting cognitive factors as hypothesized in the SSBC are shown in Figure 1.

An increasing number of studies have investigated and found support for the predictors proposed in the SSBC both in their associations with the intentions and in their stage specific differences across a range of environmentally related behaviors (Blitz et al., 2020; Blitz, 2021; Kirschner and Lanzendorf, 2020; Kowalska-Pyzalska and Byrka, 2019; Mack et al., 2019; Mei et al., 2024; Pan et al., 2021; Schroter et al., 2022). To date, four of these have specifically investigated and supported aspects of the SSBC in relation to meat consumption, specifically (Klöckner, 2017; Klöckner and Ofstad, 2017; Sunio et al., 2018; Weibel et al., 2019) and vegetarianism, more generally (Winkelmair and Jansen, 2023). For instance, in keeping with the SSBC, personal norms to reduce beef consumption predicted a goal intention to reduce beef consumption (Klöckner, 2017) and with being in a more advanced stage of behavior change for reducing meat consumption (Weibel et al., 2019). Further, a red meat reduction goal intention was associated with behavioral intentions for different reduction strategies and the goal intention was stronger for those beyond the Predecisional stage (Klöckner, 2017). Furthermore, self-reported beef consumption showed the expected patterns across the stages, with consumption significantly lower for those in the Postactional stage compared to those in the other stages. More broadly, personal norms for vegetarian meals were associated with a goal intention to eat more vegetarian meals and positive affect attitudes toward vegetarian meal were higher at the more advanced stages of change (Winkelmair and Jansen, 2023). As such, there is evidence for the SSBC being a suitable explanatory model for investigating stages of behavior change in relation to meat consumption.

These stage-specific associations and differences are considered to have implications for developing stage-tailored interventions for pro-environmental behaviors, generally (Bamberg and Schulte, 2020; Blitz et al., 2020; Klöckner, 2020; Mack and Tampe-Mai, 2016; Nachreiner et al., 2015; Sunio et al., 2017) and for meat reduction specifically (Bamberg, 2013b; Klöckner and Ofstad, 2017; Sunio et al., 2018). For instance, Klöckner and Ofstad (2017) demonstrated that an experimental group with stage-tailored information showed significantly greater stage progression over time compared to the control group who received no information and to the second control group who received randomly assigned information. As such, there is evidence for the SSBC also being a suitable model for informing interventions for reducing meat consumption.

### 2.2 Antagonistic habits and behavior

Habits have been shown to reduce the need for conscious intentions toward a behavior for that behavior to be enacted. In other words, strength of habit has typically been found to negatively moderate the positive relationship between intention and behavior (Gardner et al., 2020). However, it is important to make the distinction that, to date, many studies that have investigated habit strength, in relation to intentions and behavior, have done so for the *current* behavior negatively moderating the relationship between intentions for the *current* behavior and engagement in the *current* behavior (Gardner et al., 2020). This approach has been to investigate the role of habits in reducing the need for conscious intentions to engage in a behavior. However, behavior *change* means that there will be *new* intentions for the *new* behavior that differs to the current habitual behavior. Indeed, striving for this new behavioral goal is likely to require the suppression of existing behaviors that are antagonistic to the intended new one. Suppressing existing antagonistic behaviors is expected to be more difficult if the antagonistic behavior is a contextually-cued habit (Gollwitzer and Sheeran, 2006). In line with this, compared to priming a complimentary goal, priming an antagonistic goal has been shown to cause greater failure of an intended goal (Gollwitzer et al., 2011).

In their systematic review of behavior-prediction studies, Gardner et al. (2020) identified only five studies (of the 52 reviewed) which had investigated habits for behaviors that were *antagonistic* to the intentions they were interacted with. Of these five, four of the studies did not find that antagonistic habits also moderate the relationship between intentions and behavior.^3^ However, Thøgersen and Møller (2008) investigated the relationship between the intention to use public transport and the use of public transport. They found that the positive association between public transport intention and public transport use was weaker when a car use habit (i.e., an antagonistic habit) was stronger.

Of specific relevance the SSBC and the role of personal norms, rather than intentions, Klöckner et al. (2003) and Klöckner and Matthies (2004) investigated the role of antagonistic habits in moderating the relationship between personal norms and new behavior. In each study, they each found that strong antagonistic habits can inhibit the deliberation on personal norms, which then weakens the association between the held personal norms and the new, personal-norm-aligned behavior.

### 2.3 Habit discontinuity and life events

Habits are context dependent in that a conscious behavior that is repeated frequently enough in a stable context becomes cognitively associated with that context. The behavior can then be cued by the context without conscious intention (Phipps et al., 2024; Wood and Neal, 2007). Context can encompass the physical environment, the infrastructure, and the spatial, social and time cues within which behaviors take place (Müggenburg et al., 2015). Food choices have the potential to become habitual due to the frequency and consistency with which they are made. Indeed, Graves and Roelich (2021) concluded from a review of meat consumption behaviors and interventions that the persistence of habits presented the most significant obstacle to reducing meat consumption.

The habit discontinuity hypothesis proposes that habitual behaviors may become more vulnerable to change when the cuing context is disrupted and lost (Verplanken et al., 2008). Without the cue for automatic behavior, conscious, deliberative reflection on the behavior is required. During this period of cognitive reflection people are more open to alternative behaviors to achieve their goals (Thomas et al., 2016; Verplanken and Roy, 2016).

Context disruption may occur as a result of life events (Clark et al., 2016). Life events are episodic changes in an individual’s life that carry the potential to have significant and enduring effects for that individual’s life. Such events may trigger periods of adaptation and readjustment in an individual’s roles, responsibilities, and statuses as part of a broader life transition (Gropper et al., 2020; Hutchison, 2011). Or the events may bring changes to the physical context, such as with residential relocation, that are then associated with changes in behavior (e.g., De Haas et al., 2018; Scheiner and Holz-Rau, 2013; Whittle et al., 2022).

Although more discussion and research are required to understand the relationship between life events, habit discontinuity, and behavior change, there is evidence that the strength of individuals’ habits formed in previous contexts can weaken once they are in a new context (Walker et al., 2015). Likewise, the potential for behaviors to change following life events have been shown in relation to diet and meat consumption, which, although not yet directly tested, gives support to the habit discontinuity hypothesis. For instance, changes in meat consumption have been seen during pregnancy, with both increases (Crozier et al., 2009) and decreases (Forbes et al., 2018; Moura and Aschemann-Witzel, 2020; Moura et al., 2023) being observed. Likewise, following retirement, decreases (Si Hassen et al., 2017; Venn et al., 2017) and no statistically significant change (Patriota and Marques-Vidal, 2021) have also been observed.

### 2.4 Self-activation

Although there is evidence that life events are associated with changes in behavior, the mechanism behind these changes is not well understood. Indeed, context disruption does not always motivate new patterns of behavior (Chatterjee et al., 2012; Walker et al., 2015). As such, other considerations may be required to motivate a behavior change.

Verplanken et al. (2008) argued that, as antecedents to pro-environmental action (Stern, 2000; Stern et al., 1993), values^4^ may provide motivation for pro-environmental behavior (Verplanken and Holland, 2002). A disruption to context may provide an opportunity for the value to be cognitively activated, such that it is then able to influence behavior. Accordingly, they found greater pro-environmental concern was associated with a lower likelihood of using a car for commuting, but only for those who had recently relocated residence (Verplanken et al., 2008). The self-activation hypothesis, to date, has been understudied, with mixed results, prompting the need for further research (Haggar et al., 2019; Thomas et al., 2016; Whittle et al., 2022).

## 3 Conceptual framework and research hypotheses

### 3.1 Goal intentions, personal norms, and goal feasibility

**Figure 2:**
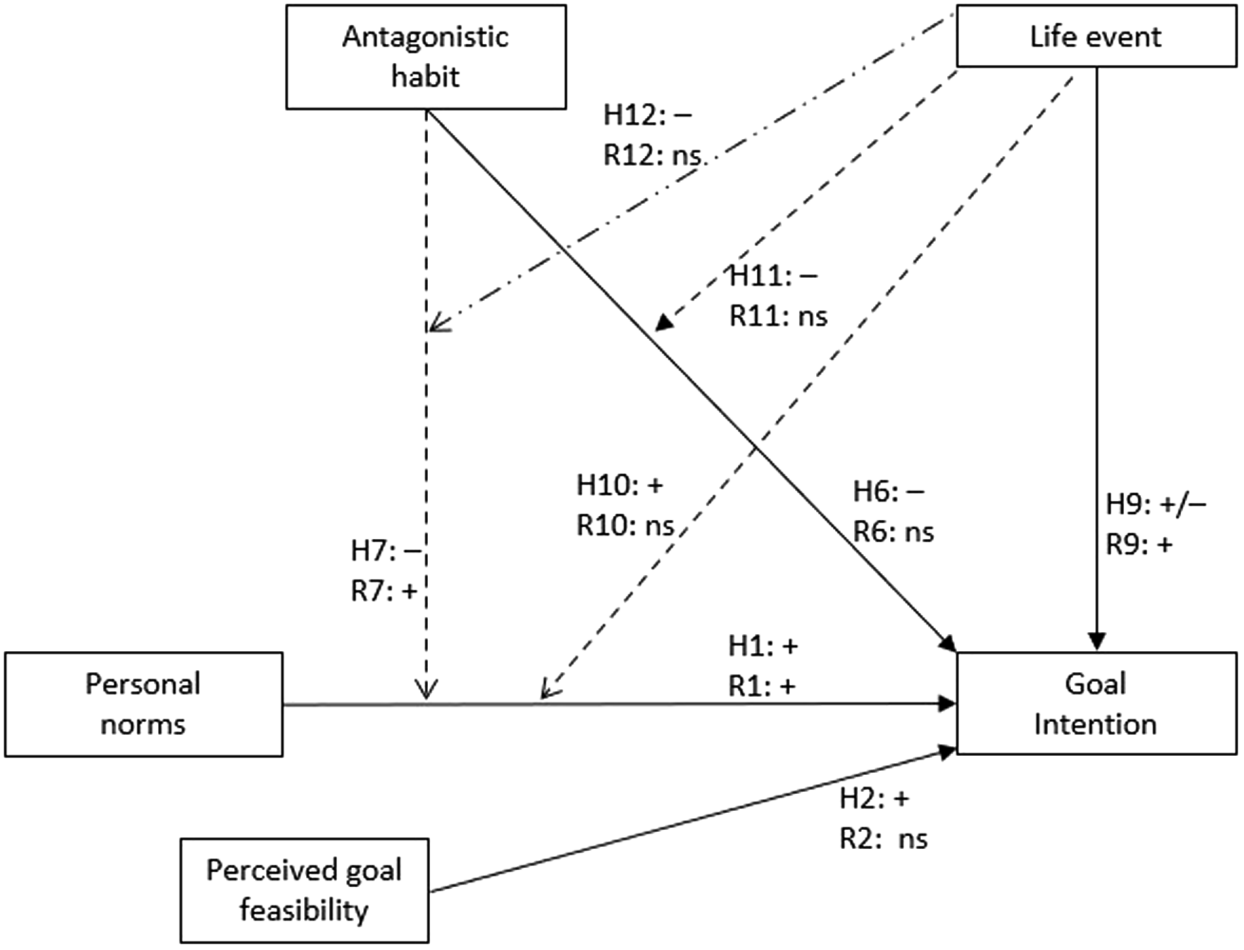
A conceptual diagram illustrating the hypothesized and found relationships between the variables of interest and goal intention. H*n* corresponds to the in-text hypotheses labeling. F*n* corresponds to the direction of the found, statistically significant relationships for that hypothesis. A positive relationship is denoted with a “+,” a negative relationship with “–,” and a non-statistically significant relationship with “ns.” Dashed lines indicate two-way interactions. The dashed and dotted line indicates the three-way interaction.

The focus of this research was on the initiation of the stages of change, the progression through the stages and how antagonistic habits and life events may promote or inhibit the factors associated with this process. As described in the reviewed literature, the formation of a goal intention is considered to indicate progression from the Predecision stage to the Preaction stage. Accordingly, of the three intentions proposed in the SSBC, the focus of this research was on the goal intention and because they are proposed as the two direct predictors of goal intentions, personal norms and goal feasibility as well. Therefore, the following hypotheses, also illustrated in Figure 2, were investigated:

*H1*: Personal norms for red meat reduction will be positively associated with the goal intention to reduce red meat consumption.

*H2*: Perceiving the goal of reducing one’s red meat consumption as more feasible will be positively related to the goal intention to reduce red meat consumption.

### 3.2 Stage membership, personal norms, and goal feasibility

**Figure 3:**
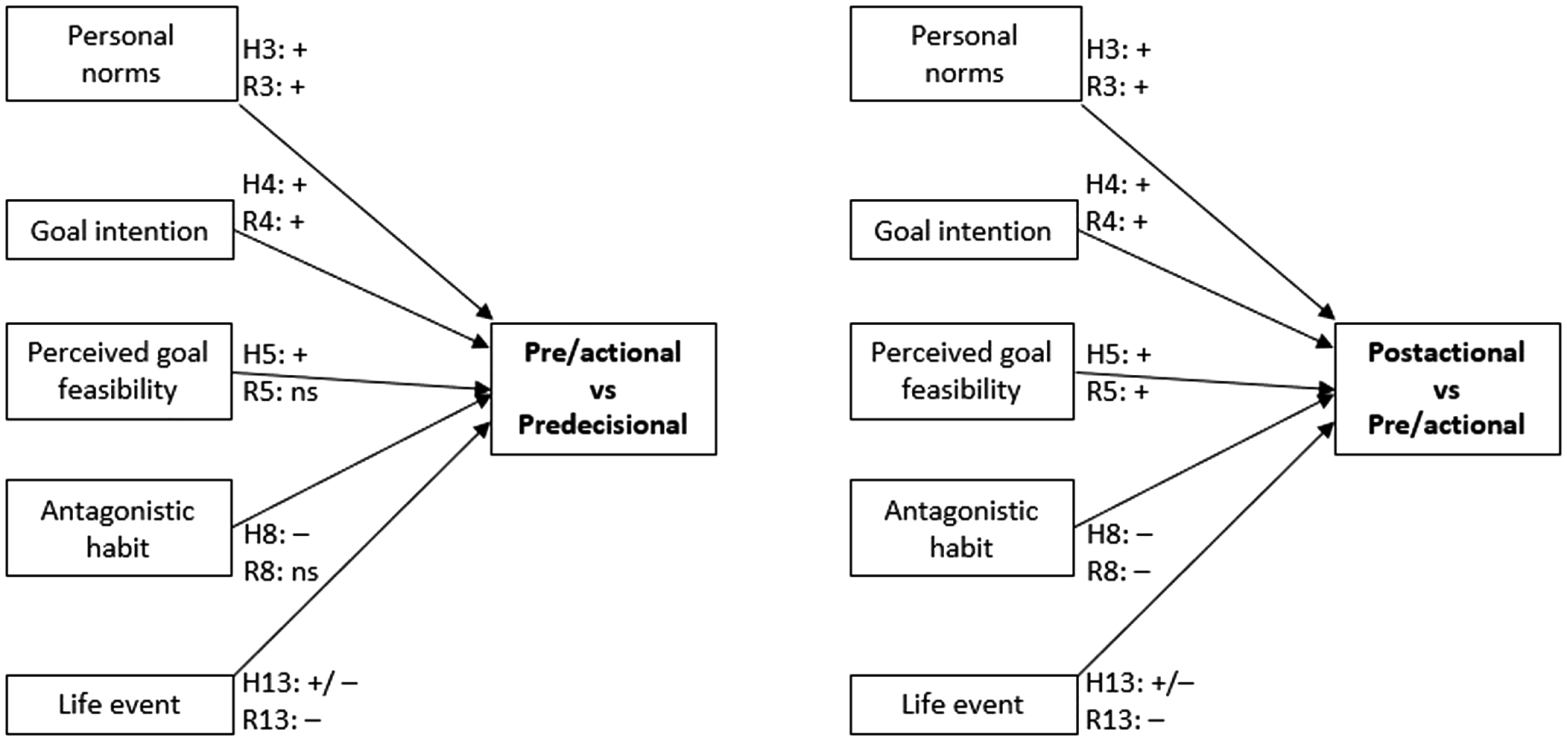
A conceptual diagram illustrating the hypothesized and found relationships between the variables of interest and the likelihood of being in the later stage vs. the earlier stage of change. A positive relationship is denoted with a “+,” a negative relationship with a “–.” H*n* corresponds to the in-text hypotheses labeling. F*n* corresponds to the direction of the found, statistically significant relationships for that hypothesis. A positive relationship is denoted with a “+,” a negative relationship with “–,” and a non-statistically significant relationship with “ns”.

As well as investigating goal intentions, we investigated stage membership, and the role that antagonistic habits and life events have in the likelihood of an individual belonging to a stage of membership beyond the Predecisional. In terms of stage membership, the SSBC proposes goal intention to be the main indicator of transitioning between the Predecisional stage and the Actional stage with personal norms and goal feasibility as its direct predictors. However, contradicting these expectations, goal intentions (Bamberg, 2013b) and personal norms (Ohnmacht et al., 2018; Weibel et al., 2019) have both been found to be associated with being in the later stages as well. Likewise, for goal feasibility has been found to relate to both goal intention (as expected) and behavioral intention (not expected; Sunio et al., 2018). As such, our hypotheses for personal norms’ (H3), goal intentions’ (H4), and goal feasibility’s (H5) relationships with stage membership did not make stage-specific predictions. The hypotheses are illustrated in Figure 3 and given below:

*H3*: Personal norms for red meat reduction will be positively associated with a greater likelihood of being in a later stage of behavior change for reducing red meat consumption.

*H4*: The goal intention for red meat reduction will be positively associated with a greater likelihood of being in a later stage of behavior change for reducing red meat consumption.

*H5*: Goal feasibility for red meat reduction will be positively associated with a greater likelihood of being in a later stage of behavior change for reducing red meat consumption.

### 3.3 Antagonistic habits and stages of change

Although the addition of the NAM factors and social norms to the MAP elucidates on the factors that contribute to the formation of a goal intention (Bamberg, 2013b; Keller et al., 2019), the contributing factors still rely on an individual becoming aware and reflecting on them before then, in turn, reflecting on their personal norms. Indeed, Gollwitzer (1990) characterized the mindset of someone in the Predecisional stage as needing to have “an open-mindedness or heightened receptivity to information” (p. 65). However, such a mindset is incongruous with habitual behavior where evidence has shown that individuals with a strong habit pay *less* attention to information about behaviors that are alternative to their habitual one (Verplanken et al., 1997). As such, when the behavior that is intended for change is habitual, individuals may not have the necessary receptivity to information about alternative behaviors to trigger a conscious reflection on their personal norms and its proposed antecedents. Indeed, Klöckner and Ofstad (2017) observed participants in the Predecisional stage sought information about reducing their beef consumption less frequently than participants in the Preactional or Actional stages. Further, as outlined, the habitual behavior may be contextually cued before any conscious deliberation on personal norms (or its proposed antecedents) can take place (Gardner et al., 2020; Klöckner et al., 2003; Klöckner and Matthies, 2004; Thøgersen and Møller, 2008).

Given the above, we argue that the current SSBC still does not fully explain how an individual engaging in a habitual (unconscious) behavior begins the Predecisional cognitive task of conscious deliberation to form their goal intention to change to an alternative behavior. Specifically, following the existing research on personal norms, intentions, and antagonistic habits (Klöckner et al., 2003; Klöckner and Matthies, 2004), and in keeping the NAM aspect of the SSBC, we argue that antagonistic habits will inhibit the activation of—and deliberation on—personal norms, which will, in turn, prevent the formation of a goal intention and so prevent the transition from the earlier to later stages of change (i.e., the Predecisional to the Preactional and so on). Accordingly, we investigate the following hypotheses for goal intention (illustrated in Figure 2) and stage of change (illustrated in Figure 3):

Strength of habit for eating red meat (i.e., antagonistic habits) will…

*H6*: …be negatively associated with goal intentions to engage in reducing red meat consumption (i.e., the new behavior).

*H7*: …negatively moderate the relationship between personal norms for reducing red meat and the goal intention for reducing red meat consumption.

*H8*: …will be negatively associated with the likelihood of being in an advanced stage of behavior change for reducing red meat consumption.

### 3.4 Self-activation, goal intention, and stage of change

As outlined, SSBC studies have demonstrated that activation of personal norms and subsequent stage progression can be induced through an intervention (Klöckner and Ofstad, 2017; Sunio et al., 2018). However, people sometimes change their behaviors without an intervention. Developing on the discussion of Verplanken and Roy (2016), and considering the habit discontinuity hypothesis and the self-activation hypothesis^5^ we argue that the experience of a life event can disrupt existing antagonistic habits, thereby providing the opportunity for deliberative reflection on held personal norms for an alternative behavior and so promote the formation of a goal intention for that behavior. Further, due to the potential for life events to disrupt antagonistic habits, the experience of a life event may also reduce any inhibition of antagonistic habits on goal intention formation and on the relationship between personal norms and goal intention. Accordingly, we hypothesize that, compared to not experiencing a life event, experiencing a life event will…

*H9*: …be associated with the goal intention for reducing red meat consumption.

*H10*: …positively moderate the relationship between personal norms for reducing red meat consumption and the goal intention for reducing red meat consumption.

*H11*: …negatively moderate the relationship between the strength of habit for eating red meat and the goal intention for reducing red meat consumption.

*H12*: …negatively moderate the strength of habit for eating red meat’s (hypothesized, H7) negative moderation of the relationship between personal norms for reducing red meat consumption and the goal intention for reducing red meat consumption.

*H13*: …be associated with the likelihood of being in an advanced stage of behavior change for reducing red meat consumption.

## 4 Method

### 4.1 Participants and procedure

To test the developed hypotheses, a cross-sectional study was conducted. The data analyzed in the present study is taken from the 12 questions provided in Supplementary Appendix A and described below. These questions formed part of a larger survey on red meat-eating attitudes and behavior totaling 36 questions. The survey was administered using online survey software. Participants were recruited through the participant panel website, Prolific.^6^ As the research questions related to reducing red meat consumption, the survey was only advertised on the website to those who had indicated to Prolific that they “do not follow a vegetarian, pescatarian, vegan, or raw food diet.” This was to minimize the likelihood that the participants were already not eating red meat. The Prolific panel consists of people who are 18 years or older and the survey was advertised to UK panel members only. Through Prolific, participants were able to access the online survey and complete it in their own time. Participants were paid for their time.

### 4.2 Measures

#### 4.2.1 Socio-demographics

Age was measured on a continuous scale. Categorical scales were used for self-identification of gender (Female, Male, Neither of the above, Prefer not to say) and presence/absence of a disability or long term health condition that affected diet (Yes, No, Prefer not to say). There were six categories for highest qualification which, for analysis, were collapsed into a binary variable of “university degree or above” and “Up to A-level.” There were six pre-tax household income categories which were also collapsed into a binary variable of “Up to £34,999” and “£35,000 or above” for analysis.

#### 4.2.2 SSBC measures

As in many of the SSBC studies to date, single item measures for goal intention for reducing red meat and goal feasibility of reducing red meat were used (Bamberg, 2013b; Keller et al., 2021; Klöckner, 2014; Ohnmacht et al., 2018; Olsson et al., 2018; Schaffner et al., 2017; Weibel et al., 2019). Personal norms for reducing red meat consumption were measured using two items adapted from Klöckner (2017). These two items formed an internally consistent scale (Cronbach’s *α* = 0.91) and were averaged to provide single score for personal norms. All items were measured using a seven point Likert-scale (strongly agree—strongly disagree) which were recoded for analysis such that a higher score indicated (respectively) having a stronger goal intention, a stronger perception that the red meat reduction goal was feasible, and having stronger personal norms to reduce one’s red meat consumption.

The participant’s current stage of behavior change was ascertained using a self-assessment, categorical scale adapted from Klöckner and Ofstad (2017). There were five statements, one for each of the stages (shown in Table 2) with an additional one for the Predecisional stage, following Bamberg (2013b) and Klöckner and Ofstad (2017). The participant was asked to select the statement that best described their current situation.

**Table 2:** Item wording for the stages of behavior change measure.

Stage of change	Item wording
Predecisional: no interest	I am satisfied with how much red meat I eat at the moment and see no need to change it
Predecisional: impossible	I would like to reduce how much red meat I eat, but at the moment I feel that this is impossible for me
Preactional	I would like to reduce how much red meat I eat, but at the moment, I am unsure about how to do so
Actional	I would like to reduce how much red meat I eat. I know how I can reduce it, but I have not put it into practice
Postactional	I am currently reducing how much red meat I eat

#### 4.2.3 Behavioral automaticity

To measure the strength of behavioral habit, the self-reported behavioral automaticity index (SRBAI; Gardner et al., 2012) was adapted to relate to buying products that contain red meat at the supermarket. Responses were recoded for analysis so that a higher score indicated stronger automatic behavior for buying products containing red meat when at the supermarket. The four items formed an internally consistent scale (Cronbach’s α = 0.95) and were averaged to provide a single score for the antagonistic habit.

#### 4.2.4 Life events experienced in the last 2 months

To ascertain if the participants had experienced at least one life event in the 2 months prior to taking the survey, we formed a list of 14 life events taken from those commonly investigated in the literature, including residential relocation, having a child, retiring, co-habitation, changes in job status, and changes in social groups (Larouche et al., 2020; Whittle et al., 2022). We deliberately avoided topics likely to be associated with negative emotions, such as bereavement to avoid undue upset for the participants.

Participants could indicate “Yes,” “No,” or “Prefer not to say” for each life event in response to whether they had experienced that event in the last 2 months. From these, a single variable “experienced a life event” was created for analysis. Those with “No” responses to *all* the life events were given a “No” on the “experienced a life event” variable. Those with a single “Yes” on any *one* life event item were given a “yes” on the “experienced a life event” variable. Those who indicated a combination of “no,” “prefer not to say” or no response across all the items were not given a value on the “experienced a life event” variable as we could not be certain if they had experienced at least one life event or not.

#### 4.2.5 Sample

In total, 406 responses were collected. As the study focus was on reducing red meat consumption, the sample of interest were those who ate red meat. As such, the 31 responses that indicated they did not eat red meat were removed from the data set. The construction of the life event variable generated missing data for 6 participants (i.e., those with the combination of “no,” “prefer not to say” or had a missing value across the items). All items had missing data of less than 5% (Supplementary Appendix B). Excluding those with missing item data when it is <5% of responses is considered unlikely to bias analyses (Dong and Peng, 2013; Schafer, 1999), further a Little’s MCAR test indicated that the missing data for the continuous variables (age, personal norms, goal intention, goal feasibility, and the SRBAI items) were missing completely at random (χ^2^(15) = 15.09, *p* = 0.52). As such, missing data was treated with exclusion as the potential for introducing bias into the analyses was deemed to be small. Overall, 20 (5.3%) participants were excluded for having missing data on one or more of the independent and/or dependent variables giving an analyzed sample of 355.

The mean age of the sample was 41.89 (SD = 14.95, range = 64) with 235 identifying as female and 120 identifying as male. In terms of education and income, 162 had formal education up to A-levels, while 193 had formal education of a degree or further and 162 had pre-tax household income of up to £34,999 and 193 had a pre-tax household income of £35,000 or above. There were 33 who indicated that they experienced a long-term disability or health condition that impacted on the food they could eat. The mean personal norms score was 3.32 (SD = 1.72), the mean goal intentions to reduce red meat consumption in the next 2 months was 3.22 (SD = 1.70), the mean perceived feasibility of the goal was 3.89 (SD = 1.44), and the mean strength of automaticity (antagonistic habit) for purchasing products that contain red meat was 4.22 (SD = 1.72). As the scale midpoints were 4, these means suggest that, on average, the participants did not have strong personal norms or goal intentions for reducing red meat, perceived the goal as a little difficult, and had somewhat strong habits for purchasing products contain red meat. Lastly, 127 had experienced at least one of the 14 specified life events in the last 2 months and 228 indicated that they had experienced none of them.

The highest frequency stage membership was the “Predecisional: no interest” stage, indicating that the majority of the sample did not feel a need to reduce the amount of meat they were eating. Following Bamberg (2013b) and Klöckner and Ofstad (2017), we combined the Predecisional: no interest and Predecisional: impossible stages into one Predecisional stage. Further, due to the frequencies in the Preactional and Actional stages being particularly low, for analysis, these stages were combined into one stage and labeled Pre/Actional. The frequencies of the subsequent three category version of the stages of change variable are shown in Table 3.

**Table 3:** Frequency of stage membership.

All stages	Predecisional: no interest	Predecisional: impossible	Preactional	Actional	Postactional
Frequency	237	23	19	33	43
Collapsed stages	Predecisional	Pre/Actional	Postactional		
Frequency	260	52	43		

#### 4.2.6 Analyses

A hierarchical, linear regression (two-tailed, alpha = 0.05) with bias corrected accelerated bootstrapped 95% confidence intervals (10,000 bootstrap iterations) was used to investigate the predictors of goal intention in three models. The first model contained the main effects of personal norms (H1), perceived goal feasibility (H2), antagonistic habit (H6), and life event (H9). The second model had the addition of a two-way interaction between personal norms and antagonistic habits (H7), a two-way interaction between life events and personal norms (H10), and a two-way interaction between life events and antagonistic habits (H11). The third model had the addition of a three-way interaction between personal norms, life events, and antagonistic habits (H12).

For the prediction of stage membership, we considered the stage variable to be ordinal in nature and therefore selected a class of ordinal regression. The forward continuation ratio model with a logit link allowed the investigation of the probability of participants advancing *beyond* their stage of behavior change, given that they have already reached their current stage of behavior change (Bürkner and Vuorre, 2019; O'Connell, 2006). Personal norms (H3), goal intention (H4), perceived goal feasibility (H5), antagonistic habit (H8), and life event (H13) were entered as predictors.

All analyses were completed with R (version 4.4.1; R Core Team, 2024) using the lm function and boot package (Canty and Ripley, 2024) for the linear regressions and the VGAM package (Yee, 2024) to fit and compare the forward continuation ratio models.

## 5 Results

### 5.1 Predicting goal intention

The *R*^2^ and the *ΔR*^2^ for each of the hierarchical models are given in Table 4. Overall, each model explained a statistically significant proportion of variance in the goal intention to reduce red meat. The addition of the two-way interaction terms significantly increased the amount of variance explained, however, the addition of the three-way interaction term did not. The available inferences from the coefficients did not change in meaning and so, for succinctness, only the results of model 3 are presented in Table 5, with the results of models 1 and 2 available in Supplementary Appendix C. The directions of the relationships are also illustrated in Figure 2.

**Table 4:** The *R*^2^, adjusted *R*^2^, and *R*^2^ change values for each model predicting the goal intention to reduce red meat consumption.

Model	*R* ^2^	Adjusted *R*^2^	*R*^2^ change
1	*F*(9, 345) =36.27, *p* < 0.001, *R*^2^ = 0.49	0.47	–
2	*F*(12, 342) = 30.00, *p* < 0.001, *R*^2^ = 0.51	0.50	*F*(3, 342) = 6.20, *p* < 0.001, *ΔR*^2^ = 0.02
3	*F*(13, 341) = 27.65, *p* < 0.001, *R*^2^ = 0.51	0.50	*F*(1, 341) = 0.31, *p* = 0.58, *ΔR*^2^ = <0.01

N.B. Statistics in this table estimated without bootstrapping. All values in this table rounded to two decimal places.

**Table 5:** Estimates from models predicting goal intention for reducing red meat consumption with life events and antagonistic habits as moderators.

	Coefficient (B)	Standard error	Lower 95% confidence interval	Upper 95% confidence interval
Intercept	0.71	0.63	−0.50	1.98
Health	0.05	0.24	−0.40	0.54
Gender	−0.26	0.13	−0.52	0.01
Age	<0.01	<0.01	<−0.01	0.01
Education	0.08	0.13	−0.18	0.35
Income	−0.02	0.15	−0.30	0.28
Personal norm for red meat reduction	0.62	0.06	0.50	0.72
Perceived goal feasibility	0.08	0.06	−0.03	0.18
Antagonistic habit	0.02	0.05	−0.09	0.12
Life event	0.31	0.14	0.03	0.59
Personal norms × antagonistic habit	0.07	0.03	0.02	0.13
Personal norms × life event	0.10	0.08	−0.07	0.25
Life event × antagonistic habit	0.08	0.10	−0.11	0.29
Personal norms × life event × antagonistic habit	0.02	0.06	−0.09	0.15

N.B. All values in this table rounded to two decimal places. Coefficients are unstandardized. Personal norms and antagonistic habits were mean centered for the interaction terms.

Personal norms for red meat reduction and experiencing a life event were significantly positively related to the red meat reduction goal intention. This indicates that those with stronger personal norms for red meat reduction also had stronger goal intentions for red meat reduction. Likewise, compared to those who had not experienced a life event, those who reported having experienced a life event had stronger goal intentions for red meat reduction. The other variables of interest, perceived goal feasibility and antagonistic habit for purchasing products that contain red meat were not significantly related to goal intentions to reduce red meat.

Contrary to our hypotheses for the two-way interactions, the personal norms and experiencing a life event interaction and the experiencing a life event and antagonistic habit interaction were not significant. While the interaction between personal norms and behavioral automaticity was significant, it was in a positive direction, not negative (as hypothesized) suggesting that those with stronger antagonistic habits had stronger positive relationships between their personal norms and goal intentions.

The three-way interaction between experiencing a life event, personal norms, and behavioral automaticity was not statistically significant.

### 5.2 Predicting stage of change membership

Initially, a proportional odds version of the forward continuation ratio model was fitted to the data wherein estimates for the predictors were constrained to equality across the stages. The parallel odds assumptions were then checked by fitting a non-proportional odds model where all estimates were allowed to vary between the stages. The estimates of these two models are provided in Supplementary Appendix D (Supplementary Tables D1, D2, respectively). The goodness of fit of each model was compared on the AIC, BIC, and with a likelihood ratio test. The AIC indicated that the non-proportional odds model (349.89) was a better fit than the proportional odds model (366.89). However, the BIC indicated that the non-proportional odds model (396.35) was a marginally worse fit than the proportional odds model (394.00). The likelihood ratio test showed that there was a significant difference between the fit of the models (χ^2^(5) = 27.00, *p* < 0.001). Overall, this was taken to indicate that the proportional odds assumption could not be met.

The potential for a partial model was then investigated by systematically freeing each predictor’s estimate individually and comparing their model fit with the proportional odds model fit. The AIC, BIC, and likelihood ratio tests for each model comparison are given in Supplementary Appendix D, Supplementary Table D3. The intercepts were free to vary in all models. It was found that the model fit improved when goal intention, goal feasibility, and behavioral automaticity were free to vary across the stages.

A partial model was then fit to the data with the estimates for the intercepts, goal intention, goal feasibility, and behavioral automaticity free to vary across the stages while the other predictors were constrained to equality. The fit of this partial model was compared to the fit of a null (intercept only) model and found to be a significantly better (χ^2^(8) = 215.41, *p* < 0.001). Finally, the fit of the partial model was compared to the fit of the non-proportional model and no significant difference was found (χ^2^(2) = 1.96, *p* = 0.38) indicating that the greater complexity of the non-proportional model did not significantly improve the fit over that of the less complex, more parsimonious, partial model and so we accepted the partial model. The estimates and odds ratios, and their respective confidence intervals, of this partial, forward continuation ratio model are shown in Table 6, with the direction of the relationships illustrated in Figure 3.

**Table 6:** Estimates and odds ratios (OR) for the partial, forward continuation ratio model with logit link and Likelihood Ratio test statistics and their respective Likelihood/profile based confidence intervals (CI).

	Estimate	*p*	Lower 95% CI	Upper 95% CI	OR	Lower 95% CI for OR	Upper 95% CI for OR
Intercept 1	–	–	−8.49	−4.65	<0.01	<0.001	0.01
Intercept 2	–	–	−9.71	−2.39	<0.01	<0.001	0.09
Personal norms	0.28	0.01	0.06	0.50	1.32	1.07	1.65
Goal intention 1	1.11	<0.001	0.83	1.42	3.03	2.29	4.15
Goal intention 2	0.66	<0.01	0.16	1.27	1.94	1.17	3.55
Goal feasibility 1	0.07	0.60	−0.20	0.35	1.08	0.82	1.41
Goal feasibility 2	0.82	<0.001	0.39	1.30	2.27	1.47	3.69
Behavioral automaticity 1	0.02	0.87	−0.20	0.23	1.02	0.82	1.26
Behavioral automaticity 2	−0.59	<0.001	−0.96	−0.26	0.56	0.38	0.77
Life events	−0.76	0.01	−1.37	−0.17	0.47	0.25	0.85

1 = Predictors’ partial estimates for the Predecisional vs Pre/Actional category membership; 2 = Predictors’ partial estimates for the Pre/Actional vs Postactional membership. Variables that are not numbered, with either “1” or “2”, had their estimates constrained to equality.

Interpreting the odds ratios (see Table 6), the results of the partial, forward cumulative ratio model indicate that personal norms for reducing red meat consumption had a significant positive association with stage progression that was equal across the stages. This means that stronger personal norms were associated with greater odds of being in the relatively higher stages, i.e., in the Pre/Actional instead of Predecisional and in the Postactional instead of Pre/Actional. Life events also had an equal association with the stages, however, it was negative, and so experiencing a life event was associated with reduced odds of being in the relatively higher stages.

In terms of stage specific associations, compared to those with weaker goal intentions, those with stronger goal intentions for reducing red meat consumption had higher odds of being in the later Pre/Actional stage than in the earlier Predecisional stage. Likewise, they had higher odds of being in the later Postactional stage then to being in the Pre/Actional stage. Goal feasibility was not associated with any difference in the odds of being in the Pre/Actional stage compared to the Predecisional. However, compared to those who believed that the red meat consumption reduction goal was not as feasible for them, those who more strongly believed it was feasible for them had greater odds of being in the Postactional stage than in the Pre/Actional stage.

In terms of the investigation of the role of antagonistic habits in behavior change stage progression, the findings also show stage specific associations. Greater behavioral automaticity for buying products that contain red meat when at the supermarket was *not* significantly associated with a difference in odds of being in the Pre/Actional stage compared to the Predecisional stage. However, compared to those who indicated weaker behavioral automaticity for buying products that contain red meat when at the supermarket, those who indicted stronger behavioral automaticity for buying products that contain red meat when at the supermarket had *reduced* odds of being in the Postactional stage compared to the Pre/Actional stage.

## 6 Discussion

In the Predecisional stage, existing behavior is often habitual (Bamberg, 2013a). However, the consequences of the behavior being habitual for the initiation of—and progression—through stages of change have only had recent attention (Mei et al., 2024) and the potential for their disruption from life events has not yet been investigated. We hypothesized that habitual behaviors that are antagonistic to the new, alternative behavior, would inhibit goal intention formation and, by extension, stage progression. At the same time, and in line with habit discontinuity (Verplanken and Roy, 2016), we hypothesized that life events may disrupt the contextual cues of the habitual behavior and in doing so, provide an opportunity for conscious deliberation on alternative behaviors that are more congruent with personal norms. This would then promote the formation of a goal intention and by extension, progression through the stages of change.

Contradicting our hypothesis (H6), but in line with Mei et al. (2024), we did not find significant a negative association between antagonistic habits and goal intention. However, we found that the antagonistic habit *positively* moderated the relationship between personal norms and goal intention. Although the positive nature of the relationship contradicts our hypothesis (H7), it does relate to findings from Semenescu and Gavreliuc (2019), who found that those with stronger car use habits expressed greater intentions to reduce their car use than those with weaker car use habits. Further, Eriksson et al. (2008) found that reductions in car use were greater for those who had stronger personal norms for car reduction *and* stronger (antagonistic) car use habits. As such, we draw on the explanation of Eriksson et al. (2008) and suggest that, through answering questions about their red meat consumption in the survey, those with the stronger antagonistic habits—and so potentially greater frequency of meat purchasing—may have become aware of the discrepancy between their high frequency meat consumption and their personal norms for reducing meat consumption. Then, because of deliberating on this discrepancy, formed a stronger goal intention to change compared to those whose personal norms and behavior were already more aligned (i.e., weaker antagonistic habits not as discrepant with personal norms).

Considering the role of life events in goal intention formation (H9), compared to those who had not experienced a life event, those who had experienced a life event had a stronger goal intention for reducing red meat. However, the non-significant interaction between life events and personal norms does not support our self-activation hypothesis (H10). Perhaps, in this instance, the personal norms for red meat reduction were already so strongly related to the goal intention to reduce red meat consumption that experience of a life event was not necessary for cognitively activating personal norms and strengthening goal intentions. The non-significant interaction between life event and antagonistic also habit does not support our habit discontinuity hypothesis (H11). Finally, despite antagonistic habits *positively* moderating the relationship between personal norms and goal intentions, we would still have expected the experience of a life event to have, in turn, negatively moderated the antagonistic habits’ moderation due to the habit being disrupted. The non-significant three-way interaction, however, did not support our hypothesis (H12). As such, although our findings show that the experience of a life event is positively associated with goal intention formation, the mechanism for this association is still not clear from our findings.

In terms of the stage membership findings and the SSBC factors, our results support our hypotheses (H3 and H4) by showing that personal norms and goal intention are important for transitioning from the Predecisional to the Pre/Actional stage and from the Pre/Actional to the Postactional stage. Goal intention, however, was found to diminish in importance for the transition between the Pre/Actional and Postactional stages, which would be expected for the SSBC concept and has been found in previous investigations of SSBC stage membership (Bamberg, 2013b). However, in contradiction with the SSBC concept, personal norms were found to be *equally* important for each of the stage transitions. Although this equality contradicts the SSBC, it is in line with Weibel et al. (2019) and suggests that personal norms continue to have a role beyond the Predecisional cognitive task of deliberation and are also involved in the Pre/Actional cognitive tasks of planning and engaging in the behavior. As there is evidence that personal norms can be reinforced through engaging in aligned behavior (Thøgersen and Ölander, 2006), we might expect progression through the stages of change to feedback and strengthen personal norms for the new behavior, which could explain the continued importance of personal norms.

Partially supporting our hypothesis (H5), perceived goal feasibility, was not important for the transition from the Predecisional to the Pre/Actional stage, but it was important for transitioning from the Pre/Actional stage to the Postactional stage. This suggests that perceived goal feasibility may be less important for the Predecisional cognitive task of deliberating behavior change behavior than it is for the Pre/Actional cognitive tasks of planning and engaging in the behavior. This would contradict the purpose of goal feasibility as proposed in the SSBC (Bamberg and Schulte, 2020) and so further research on goal feasibility is needed to replicate our findings.

Overall, these findings for the SSBC factors support the utility in supporting activation of personal norms and goal intention formation in stage-targeted behavior change interventions (Bamberg and Schulte, 2020; Blitz et al., 2020; Klöckner, 2020; Mack and Tampe-Mai, 2016; Nachreiner et al., 2015; Sunio et al., 2017). They additionally suggest that those in the Preactional and Actional stages may continue to benefit from personal norm activation and may also benefit from support in increasing the perceived feasibility of their intended behavior.

For the role of life events in stage models of behavior change, our findings suggest life events may have inhibited progression toward behavior change. This supports our hypothesis (H13) and demonstrates the value of investigating life events with stages of behavior changes. Indeed, our findings with the stage model, offer insight into how life events relate to the *pursuit* of behavior change following a life event. It helps in understanding how to bridge the gaps between intentions and behavior (ElHaffar et al., 2020) by breaking down the latent psychological changes that follow intention and precede behavior change. As such, as an explanation of our findings, we consider that, while life events may be able to disrupt habits and promote conscious deliberation, they can also be subjectively stressful experiences. Stress has been shown to increase avoidance of cognitive effort (Bogdanov et al., 2021) and so stress may cause avoidance of the cognitively effortful tasks of behavior change. As such, a stressful life event maybe impede behavior change progression, even if existing habits are disrupted. Accordingly, we propose that individual differences in psychological resilience may be an important psychological factor in determining the behavioral outcomes of the life event experience for the individual (Fletcher and Sarkar, 2013; Troy et al., 2023) and may act to moderate the relationship between life events and stage progression.

The negative relationship between life event and stage membership appears to contradict our finding of a *positive* relationship between life event and goal intention to reduce red meat consumption. However, the difference may be indicative of the fact that the stage of change items related to the respondents’ *current* stage whereas the goal intention item referred to the respondents’ change intention for the *next* 2 months. This provides an interesting and novel insight into the life events and behavior literature by suggesting that (on average) the experience of a life event impedes behavior change, but after a prompt for goal setting (as given in the study), the experience of a life event promotes stronger goal intentions for change. Combined with the continued mixed findings for the self-activation hypothesis (Haggar et al., 2019; Thomas et al., 2016; Verplanken et al., 2008; Whittle et al., 2022), to which our results add another non-significant result, these life event findings could suggest that behavior change following a life event may need external prompting. However, the investigation of self-activation, to date, has varied greatly in its operationalization and methods used and so investigation into the mechanisms for how behaviors change in relation to life events without external prompting from interventions is still warranted. Indeed, our findings do still support the importance of personal norms for behavior change progression.

The antagonistic habit also had negative associations with stage membership, but they were stage-specific. In partial contradiction to H8, antagonistic habit strength was *not* significantly associated with a difference in odds of being in the Pre/Actional stage(s) compared to the Predecisional stage. However, compared to those with weaker antagonistic habits, those who indicted stronger antagonistic habit strength had *reduced* odds of being in the Postactional stage compared to the Pre/Actional stage(s). As such, our results suggest that antagonistic habits may not inhibit the *decision* to change behavior (i.e., formation of a goal intention and transition into the Preactional stage) as we hypothesized, but they could inhibit deciding *how* and *when* to engage in the new behavior, thus inhibiting the transition from the Preactional and Actional stages into the Postactional stage. Indeed, this is supported by Mei et al. (2024) who found antagonistic habits were not significantly associated with goal intentions however, they were negatively associated with behavioral intentions (the intentions necessary to transition from the Preactional to the Actional stage). As such, it is plausible that antagonistic habits may increase the likelihood of being in the attitude-intention-behavior gap as an individual may possess the goal intention to reduce their meat consumption, but their antagonistic habit inhibits any further deliberation or engagement in the actual behavior change process (i.e., progressing past the Preactional and Actional stages).

### 6.1 Implications

As the first study to investigate antagonistic habits and life events in a stage model of behavior change, our findings offer important theoretical and practical insights. Critically, our findings not only support the view that behavior change interventions need to be tailored to an individual’s current stage of change (Bamberg, 2013a; Klöckner and Ofstad, 2017; Sunio et al., 2018), but, as life event experience was associated with stronger goal intentions to change, our findings also support the strategy of delivering behavior change interventions to individuals who are experiencing context disruption (Bamberg, 2006; Verplanken and Roy, 2016). Consequently, we suggest that there is a potential to *combine* the stage-tailored intervention strategy with the strategy of targeting periods of context disruption. As noted by Klöckner and Ofstad (2017), even for stage-tailored interventions, it can be challenging to get those who are in the Predecisional stage to engage with an intervention, particularly if the intervention relies on an individual’s self-motivated information seeking or giving attention to ambient information. However, as discussed, context disruption brought about by life events may present an opportunity for individuals to deliberate on new behaviors and to act in line with their personal norms without the need for external interventions. Indeed, despite our non-significant antagonistic habit results from this study, we would still expect antagonistic habits to inhibit progress beyond the Predecisional stage and encourage further investigation of antagonistic habits in this and all aspects of stage models. However, our current findings suggest that antagonistic habits do inhibit Postactional stage membership, and so we argue that stage-targeted interventions for those in the Preactional or Actional stages of behavior change could be augmented by including features that address the breaking of existing antagonistic habits. As such, we argue that tailoring behavior change interventions to not only what stage of change the individual is currently in, but *also* to the current state of their context (e.g., have they recently experienced a life event?) could create powerful interventions for behavior change.

### 6.2 Limitations and future studies

The lack of support for the habit discontinuity hypothesis (H11 and H12) may be due to the drawback of our cross-sectional design. There is debate, but not much evidence currently, about how long a habit may stay disrupted for following a context change (sometimes referred to as the “window of opportunity”) (Adjei and Behrens, 2023; McMillan et al., 2023; Moura et al., 2023) or indeed, how long habit strength is retained following a context disruption (Walker et al., 2015). We asked participants to report life events from up to 2 months ago. As such, in our sample, antagonistic (red meat purchasing) habits may have already been re-established following the life event, or indeed, not yet been broken. Consequently, the experience of a life event was potentially either no longer having any effect on the antagonistic habits or yet to have had one. This highlights the importance of longitudinal research for life event and habit disruption investigations, akin to Eriksson et al. (2008) intervention. The cross-sectional nature of our data also prevented a full investigation of stage progression (or regression) and further research should investigate stage membership over time in general (Keller et al., 2019; Klöckner, 2014), but also specifically in relation to the role of antagonistic habits and life events as our findings suggest they can have a role in stage models of behavior change.

The data in the present study had a higher proportion of participants who identified as female compared to male and there were slightly higher proportions of those with a degree or higher and of those with a pre-tax household income of over £35,000. In the UK, women have been found to eat, on average, less meat than men (Horgan et al., 2019), although, amounts of meat have been found not to significantly differ when considered as a percentage of food energy (Stewart et al., 2021). Further, in the UK, higher qualifications, although not income, have been found to be associated with a greater likelihood of vegetarianism (Gale at al., 2007). As such, the sample imbalances could have implications for the generalizability of our results to other socio-demographic groups and it would be of value to pursue the exploration of red meat consumption in a nationally representative sample, as well as cross-nationally, to reduce potential biases as much as possible.

## 7 Conclusion

Our investigation provides novel theoretical insights into the role of habits and life events in a stage model of behavior change. Our findings show that antagonistic habits and life events can inhibit progression through the stages of change, but that life events and personal norms can support the formation of a goal intention to change, which is itself associated with progression through the stages. The findings are valuable in the applied context of understanding how to reduce over-consumption of red meat (or other, habitual, greenhouse-gas intensive behaviors) by emphasizing the importance of stage tailored behavior change interventions and we suggest the potential to combine such interventions with identifying a period when existing habits are weakened due to context disruption, such as following a life event. Overall, through the findings of this study, we have begun to elucidate on the role of antagonistic habits in stage models of behavior change and have identified promising avenues of future research.

## Data Availability

The raw data supporting the conclusions of this article will be made available by the authors, without undue reservation.

## Footnotes

^1^Although our study focuses on individual-level behavioral change, we note that political and meat industry actors are also integral to achieving sustainability targets (Lazarus et al., 2021; Sievert et al., 2021).

^2^Bamberg (2013b) also drew on the Theory of Planned Behavior (Ajzen, 1991) to add further cognitive factors that could support the formation of each of the stage-specific intentions (see Figure 1). As not all factors and relationships were tested for the present study, an explanation of all the cognitive factors will not be provided here.

^3^Gardner et al. (2020, p. 15) concluded in text that *none* of the five studies supported the hypothesis that antagonistic habits can moderate the relationship between intentions and behavior. However, in their summary table of the reviewed papers, they report a significant moderation in Thøgersen and Møller (2008). Our own reading of Thøgersen and Møller (2008) found that a significant moderation was reported, which we have subsequently described.

^4^Values can be defined as: “desirable, trans-situational goals, varying in importance, that serve as guiding principles in people’s lives” (Schwartz et al., 2001 p. 521).

^5^To adopt the self-activation hypothesis, but be in keeping with the SSBC, the present study, rather than investigate the activation of values, investigates the activation of personal norms.

^6^ http://www.prolific.com
