# Supplementary material for: The inclusion of habits in the stage model of self-regulated behavior change: an investigation of life events and red meat consumption in the UK
Source: Front Psychol. 2024 Nov 7;15:1426171. doi: 10.3389/fpsyg.2024.1426171 (PMC11578820; doi:10.3389/fpsyg.2024.1426171)
Supplement: Supplementary file 1 [file Table_1.DOCX]

**APPENDICES**

**Appendix A**

The full survey as presented to participants

Note: Light grey text was *not* seen by the participants. Further, some of the formatting of this Word document version is different to that of the online software’s, but has not been corrected.

Start of Block: Informed Consent and GDPR statement

Q1
*Thank you for your interest in taking part in our survey!*
  
**Important: please read the following information before continuing**  
 **Purpose of the research**
 Everyone is different in what they choose to eat. We wish to explore these differences to understand the nature of them and consider why they might exist. We will provide more information about the study at the end.

 **What taking part involves**
 You must be 18 years or older to participate in this study. This survey will take approximately 16 minutes to complete. You will be asked a series of multiple choice questions. Very occasionally, you might be asked to enter a number.

 We will not ask for any information that could be used to identify you. As such, your answers will be anonymous. This means that you will not be identifiable in any reports, presentations, or other means of dissemination that we use following the completion of the study. During the project we will securely store the anonymous data and use it only for research purposes. At the end of the project, the anonymous data will be stored indefinitely in the UK Data Archive, a data repository website where it will be accessible to users who have registered with the UK Data Archive. Use of the repository is to add transparency and value to our research as the wider research community may validate our findings and utilise the data.

 If you do not wish to answer a question then you do not have to, you can just to move to the next question or page. The only exception is the consent question at the bottom of this page. This one will require an answer because we will need your consent to take part before you can begin.

 You can withdraw from participating in the survey at any time by closing the browser window. You will not be asked to give a reason. We will still receive your answers from the start of the survey up to the point that you close the survey. Because we will not be asking for identifying information, we will not be able to identify your individual responses in our dataset and so will not be able to remove them from our dataset. This applies to both partially complete and completed surveys.

 For our research to be meaningful, we need you to read and answer each question carefully. There are no right or wrong answers to the questions, they are just your opinions and experiences. We really do appreciate real, honest answers!

 We will be asking you to indicate whether or not you have experienced a selection of life events (such as moving house or starting a new job) in the last 2 months and their impact on your life. If you feel that thinking about any events in the last 2 months will be upsetting for you, then please do not take part.

 *Key points:* The survey should take around 16 minutes to complete. It will not be possible to identify you from your answers. The survey is anonymous. If you do not wish to answer a question, you do not have to. Just move on to the next one. You may leave the study at any time by closing your browser window. You will not need to give an explanation.               You must be 18 years or older to participate in this study.  
 **Who to contact**
 If you have any questions, concerns, or would like to speak with us about the study, please get in touch with ...
 You can also get in touch with the Principal Investigator of the study...

Our postal address is: ...

 If you have any concerns related to your participation in this study, please contact the Psychology Research Ethics Committee: ...

 **Informed consent declaration** I understand the broad nature and purpose of the study and that it is being conducted for the promotion of knowledge and research and for no other purpose. I understand that my participation in the study is entirely voluntary and that I can withdraw, without giving a reason, at any point while answering the survey (by closing the browser window). At the end of the study, I will be provided with additional information about the purpose of the study. I understand that my anonymous survey responses will be stored indefinitely with the UK Data Archive and will be accessible to those who are registered with the UK Data Archive. I understand that I am free to discuss any questions or concerns with the main researcher, Principal Investigator, or Ethics Committee through the contact details provided. The information provided will be held in compliance with the Data Protection Act 2018 (UK responses) and GDPR regulations. The University of Bath is the data controller and ... is the data protection officer. The lawful basis for processing this information under Data Protection law is that it is necessary as part of our public task as a research institution for scientific and historical research purposes, in accordance with the necessary safeguards, and is in the public interest. This information is being collected by ... and .... .The University of Bath’s privacy notice can be found at: (opens in new window).

 **Please confirm whether or not you wish to begin the survey by selecting the appropriate response below:**

- I confirm that I am 18 years old or older and that I have read and understood the above information. I give my consent to start the survey (1)
- I do not wish to start the survey (this will end the survey) (2)

End of Block: Informed Consent and GDPR statement

Start of Block: Prolific ID

| 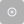 |
| --- |

Q2 What is your Prolific ID?
 *Please note that this response should auto-fill with the correct ID*

________________________________________________________________

End of Block: Prolific ID

Start of Block: Health impact on choices

Q3
**As we will be asking about what you typically eat, we would like to take into account any health considerations you have that impact on your choices. Please answer the following:**
 
Do you consider yourself to have a disability or long-term health condition that impacts on what foods you can eat?

- Yes (1)
- No (2)
- Prefer not to say (3)

End of Block: Health impact on choices

Start of Block: Socio-demographics

Q4 We are starting with just a few questions about you. As with all the questions, these questions will not identify you to us (i.e. the survey will remain anonymous), but they will help us understand your answers and ensure that we are hearing from as wide a variety of people as possible.

Q5 How do you self-identify?

- Female (1)
- Male (2)
- Neither of the above (if you wish, please specify) (3) __________________________________________________
- Prefer not to say (4)

Q6 What is your age, in years?

|  | 0 | 12 | 24 | 36 | 48 | 60 | 72 | 84 | 96 | 108 | 120 |
| --- | --- | --- | --- | --- | --- | --- | --- | --- | --- | --- | --- |

| () | 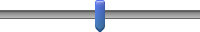 |
| --- | --- |

| Page Break |  |
| --- | --- |

| Page Break |  |
| --- | --- |

Q7 What is the highest level of formal education qualification you have completed?

- Higher Degree (e.g., Master's degree, PhD, MD, BDS) (1)
- Bachelor’s Degree (3)
- A Level, Higher Certificate, NVQ, BTEC, Baccalaureate, AS Level (4)
- GCSE, O Level, National Certificate, CSE (5)
- Other school qualification (e.g., school leaving exam certificate or matriculation) (6)
- None of the above (7)

| Page Break |  |
| --- | --- |

| Page Break |  |
| --- | --- |

Q8 What is the approximate pre-tax income of your household per year

- Below £20,000 (1)
- £20,000-£34,999 (2)
- £35,000-£49,999 (3)
- £50,000-£64,999 (4)
- £65,000-£79,999 (5)
- £80,000 or more (6)

| Page Break |  |
| --- | --- |

End of Block: Socio-demographics

Start of Block: Dietary preferences

Q9 **Out of the following foods, which do you eat? (you may select more than one)**

- Red meat (e.g. beef, lamb, pork/ham) (1)
- White meat (e.g. chicken, turkey) (2)
- Seafood (e.g. fish, shellfish) (3)
- Meat substitute products (e.g. Quorn, Linda McCartney) (4)

End of Block: Dietary preferences

Start of Block: Frequency of meat consumption

Carry Forward Selected Choices from "Out of the following foods, which do you eat? (you may select more than one)"

| 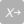 |
| --- |

Q10 **Over the last 5 days, on how many days did you (not your household) eat at least one meal or snack that contained...**

|  | Did not eat in the last 5 days (1) | 1 day (2) | 2 days (3) | 3 days (4) | 4 days (5) | 5 days (6) |
| --- | --- | --- | --- | --- | --- | --- |
| Red meat (e.g. beef, lamb, pork/ham) (x1) |  |  |  |  |  |  |
| White meat (e.g. chicken, turkey) (x2) |  |  |  |  |  |  |
| Seafood (e.g. fish, shellfish) (x3) |  |  |  |  |  |  |
| Meat substitute products (e.g. Quorn, Linda McCartney) (x4) |  |  |  |  |  |  |

| Page Break |  |
| --- | --- |

Q11 **Over the last 5 days, on how many days did you (not your household) eat at least one meal or snack that...**

|  | Did not eat in the last 5 days (1) | 1 day (2) | 2 days (3) | 3 days (4) | 4 days (5) | 5 days (6) |
| --- | --- | --- | --- | --- | --- | --- |
| Was entirely plant-based (i.e., containing no meat, fish, or dairy products). (3) |  |  |  |  |  |  |

End of Block: Frequency of meat consumption

Start of Block: Quantity of red meat consumption

Q12
*Great, thank you! This question has a longer explanation than any of the others. Please continue to read carefully.*

 We would now like to ask you about how much **red meat** (e.g. beef, lamb, pork/ham) you personally have eaten over the **last 5 days** (i.e., just you, not your whole household).   To do this, we are going to ask you to estimate the **total number** of red meat **portions** you have had.
 
We know that what constitutes a “portion” of red meat can vary from person to person. To help, we have provided some examples of **a single portion** for some common types of cooked, red meat. These examples are given in the tables below. Please **use these examples** when estimating how much red meat you have eaten in the **last 5 days**.
 
For instance, the example portion of pork sausages is two pork sausages. If you ate three pork sausages in the last 5 days, then we would like you to count that as 1.5 (one and a half) portions.

 If you ate some red meat that is not mentioned in the examples or was prepared in a way that is not shown in our examples, please use the examples as a guide to make an estimation of how many portions you had of that red meat.
 Please consider red meat in **all the meals you ate**, including snacks, food you prepared yourself, that you bought from a restaurant or from a takeaway, had cooked for you etc.

 We understand that this question might be a bit difficult, but please do give it some thought. If you are struggling to remember all your meals and portion sizes, please give your best estimate.

Q13 **Example portion sizes for red meat**
   **Beef** **Mince** Six tablespoons (100g) **Steak** One, the size of the palm of your hand (130g) **Burger** One, medium sized (80-100g)  
   **Pork** **Sausage** Two, medium sized (90g) **Ham** Two slices, thin cut (30g) **Bacon** Two slices (50g)  
   **Lamb** **Mince** 6 tablespoons (100g)

| 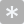 |
| --- |

Q14 Please give your estimate for the **total number** of **red meat** *portions* (e.g. beef, lamb, pork/ham) you have eaten in the **last 5 days**:

________________________________________________________________

End of Block: Quantity of red meat consumption

Start of Block: Red meat expenditure

Q15 Thinking about your **food shopping**, what *percentage* of your **monthly food bills** would you estimate comes from purchases of **red meat** (e.g. beef, lamb, pork/ham) or from products that contain **red meat** (e.g. sausages, pepperoni pizza, steak and kidney pie etc.)?

|  | 0 | 10 | 20 | 30 | 40 | 50 | 60 | 70 | 80 | 90 | 100 |
| --- | --- | --- | --- | --- | --- | --- | --- | --- | --- | --- | --- |

| () | 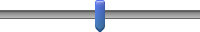 |
| --- | --- |

| Page Break |  |
| --- | --- |

Q16
Please think about how much *money* you **currently spend** on **red meat** (e.g. beef, lamb, pork/ham) or from products that contain red meat (e.g. sausages, pepperoni pizza, steak and kidney pie etc.). How does this **compare** to your spending on **red meat 2 months ago**?
 
I spend...

- A lot less than before (1)
- Less than before (2)
- About the same as before (3)
- More than before (4)
- A lot more than before (5)

End of Block: Red meat expenditure

Start of Block: Red meat buying habit -supermarket raw

Q17 **Please consider the following statements and indicate to what extent you agree or disagree with them.**


 When at the supermarket, buying raw (uncooked) red meat (e.g. beef, pork, or lamb) is something...

|  | Strongly agree (1) | Agree (2) | Somewhat agree (3) | Neither agree nor disagree (4) | Somewhat disagree (5) | Disagree (6) | Strongly disagree (7) |
| --- | --- | --- | --- | --- | --- | --- | --- |
| ...I do automatically (1) |  |  |  |  |  |  |  |
| ...I do without having to consciously remember (2) |  |  |  |  |  |  |  |
| ...I do without thinking (3) |  |  |  |  |  |  |  |
| ...I start doing before I realise I am doing it (4) |  |  |  |  |  |  |  |

End of Block: Red meat buying habit -supermarket raw

Start of Block: Red meat buying - eating out

Q18 When in takeaways, cafés or restaurants, considering meal options that contain red meat (e.g. beef, lamb, pork/ham) is something...

|  | Strongly agree (1) | Agree (2) | Somewhat agree (3) | Neither agree nor disagree (4) | Somewhat disagree (5) | Disagree (6) | Strongly disagree (7) |
| --- | --- | --- | --- | --- | --- | --- | --- |
| ...I do automatically (1) |  |  |  |  |  |  |  |
| ...I do without having to consciously remember (2) |  |  |  |  |  |  |  |
| ...I do without thinking (3) |  |  |  |  |  |  |  |
| ...I start doing before I realise I am doing it (4) |  |  |  |  |  |  |  |

End of Block: Red meat buying - eating out

Start of Block: Red meat buying - supermarket ready made

Q19 When at the supermarket, buying products that contain red meat (e.g. beef, lamb, pork/ham; e.g. pies, soups, pizzas etc.) is something...

|  | Strongly agree (1) | Agree (2) | Somewhat agree (3) | Neither agree nor disagree (4) | Somewhat disagree (5) | Disagree (6) | Strongly disagree (7) |
| --- | --- | --- | --- | --- | --- | --- | --- |
| ...I do automatically (1) |  |  |  |  |  |  |  |
| ...I do without having to consciously remember (2) |  |  |  |  |  |  |  |
| ...I do without thinking (3) |  |  |  |  |  |  |  |
| ...I start doing before I realise I am doing it (4) |  |  |  |  |  |  |  |

End of Block: Red meat buying - supermarket ready made

Start of Block: The 4 Ns of meat justification

| 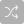 |
| --- |

Q20
Thank you!


Please consider the statements below. For each one, indicate the extent to which you agree or disagree with it:

|  | Strongly agree (1) | Agree (2) | Somewhat agree (3) | Neither agree nor disagree (4) | Somewhat disagree (5) | Disagree (6) | Strongly disagree (7) |
| --- | --- | --- | --- | --- | --- | --- | --- |
| It is only natural to eat meat (1) |  |  |  |  |  |  |  |
| Our human ancestors ate meat all the time (2) |  |  |  |  |  |  |  |
| It is unnatural to eat an all plant-based diet (3) |  |  |  |  |  |  |  |
| Human beings are natural meat-eaters –we naturally crave meat (4) |  |  |  |  |  |  |  |
| It is necessary to eat meat in order to be healthy (5) |  |  |  |  |  |  |  |
| A healthy diet requires at least some meat (6) |  |  |  |  |  |  |  |
| You cannot get all the protein, vitamins and minerals you need on an all plant based diet (7) |  |  |  |  |  |  |  |
| Human beings need to eat meat (8) |  |  |  |  |  |  |  |
| It is normal to eat meat (9) |  |  |  |  |  |  |  |
| It is abnormal for humans not to eat meat (10) |  |  |  |  |  |  |  |
| Most people eat meat, and most people can’t be wrong (11) |  |  |  |  |  |  |  |
| It is common for people to eat meat in our society, so not eating meat is socially offensive (12) |  |  |  |  |  |  |  |
| Meat is delicious (13) |  |  |  |  |  |  |  |
| Meat adds so much flavour to a meal it does not make sense to leave it out (14) |  |  |  |  |  |  |  |
| The best tasting food is normally a meat-based dish (e.g., steak, chicken breast, grilled fish) (15) |  |  |  |  |  |  |  |
| Meals without meat would just be bland and boring (16) |  |  |  |  |  |  |  |

End of Block: The 4 Ns of meat justification

Start of Block: Red meat - Current stage of behaviour change

Q21 From the options below, please choose which statement fits your current situation best:

- I am satisfied with how much red meat I eat at the moment and see no need to change it. (1)
- I would like to reduce how much red meat I eat, but at the moment I feel that this is impossible for me. (2)
- I would like to reduce how much red meat I eat, but at the moment, I am unsure about how to do so. (3)
- I would like to reduce how much red meat I eat. I know how I can reduce it, but I have not put it into practice. (4)
- I am currently reducing how much red meat I eat. (5)

End of Block: Red meat - Current stage of behaviour change

Start of Block: Goal intention to reduce red meat

Q22 Please consider the following statement and indicate the extent to which you agree or disagree with them:

|  | Strongly agree (1) | Agree (2) | Somewhat agree (3) | Neither agree nor disagree (4) | Somewhat disagree (5) | Disagree (6) | Strongly disagree (7) |
| --- | --- | --- | --- | --- | --- | --- | --- |
| I intend to reduce how much red meat I eat in the next 2 months... (1) |  |  |  |  |  |  |  |

End of Block: Goal intention to reduce red meat

Start of Block: Goal feasibility

Q23 Please consider the following statement and indicate the extent to which you agree or disagree with them:

|  | Very easy (1) | Easy (2) | Somewhat easy (3) | Neither easy nor difficult (4) | Somewhat difficult (5) | Difficult (6) | Very difficult (7) |
| --- | --- | --- | --- | --- | --- | --- | --- |
| Reducing how much red eat I am eating in the near future will be... (1) |  |  |  |  |  |  |  |

End of Block: Goal feasibility

Start of Block: Behavioural intention to reduce red meat

Q24 I have decided *how* I will reduce the amount of red meat in some or all of my meals. I intend to make a plan on when to start doing it...

- Strongly agree (8)
- Agree (9)
- Somewhat agree (10)
- Neither agree nor disagree (11)
- Somewhat disagree (12)
- Disagree (13)
- Strongly disagree (14)

End of Block: Behavioural intention to reduce red meat

Start of Block: Implementation intention to reduce red meat

Q25 I have already planned *when* I will reduce the amount of red meat in some or all of my meals...

- Strongly agree (8)
- Agree (9)
- Somewhat agree (10)
- Neither agree nor disagree (11)
- Somewhat disagree (12)
- Disagree (13)
- Strongly disagree (14)

End of Block: Implementation intention to reduce red meat

Start of Block: Personal norms, subjective norms

| 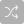 |
| --- |

Q26 Please consider the following statements and indicate the extent to which you agree or disagree with them:

|  | Strongly agree (1) | Agree (2) | Somewhat agree (3) | Neither agree nor disagree (4) | Somewhat disagree (5) | Disagree (6) | Strongly disagree (7) |
| --- | --- | --- | --- | --- | --- | --- | --- |
| Based on my personal values, I feel obliged to reduce how much red meat I am eating. (1) |  |  |  |  |  |  |  |
| Regardless of what other people do, because of my own principles, I feel an obligation to reduce how much red meat I am eating. (2) |  |  |  |  |  |  |  |
| Most of the people that are important to me would support me if I were to reduce my meat consumption. (3) |  |  |  |  |  |  |  |
| Among the different options for reducing how much red meat I eat, there is at least one which I will find easy (4) |  |  |  |  |  |  |  |
| There is at least one option for reducing red meat consumption that I have complete control over (5) |  |  |  |  |  |  |  |
| Out of the different options for reducing red meat consumption, I have thought through a plan for how to do at least one of them (6) |  |  |  |  |  |  |  |

End of Block: Personal norms, subjective norms

Start of Block: Perceived monetary costs of change

Q27
Last few about food...


Thinking about how much money you spend on eating red meat, please select one of the options to complete the sentence:
 Switching to a diet with less red meat would be...

- ...extremely cheap for me (1)
- ...a lot cheaper for me (2)
- ...cheaper for me (3)
- ...neither cheaper nor more expensive more for me (4)
- ...more expensive for me (5)
- ...a lot more expensive for me (6)
- ...extremely expensive for me (7)

End of Block: Perceived monetary costs of change

Start of Block: Perceived availability of alternatives to red meat

Q28 Thinking about the shop (or shops) where you buy most of your food, how would you rate the availability of *alternatives* to red meat (e.g. beef, lamb, pork/ham) for your meals?

- Very good (1)
- Good (2)
- Somewhat good (3)
- Neither good nor bad (4)
- Somewhat bad (5)
- Bad (6)
- Very bad (7)

| 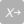 |
| --- |

Q29 Thinking about places you regularly eat-out at (e.g. restaurants, cafes, workplace canteens), how would you rate the availability of options that *do not contain* red meat?

- Very good (1)
- Good (2)
- Somewhat good (3)
- Neither good nor bad (4)
- Somewhat bad (5)
- Bad (6)
- Very bad (7)
- I never eat out (8)

Q30 Thinking about near where you live, how would you rate the availability of restaurants, cafés, and takeaways (whether you eat there yourself or not) that serve meals *without* red meat?

- Very good (1)
- Good (2)
- Somewhat good (3)
- Neither good nor bad (4)
- Somewhat bad (5)
- Bad (6)
- Very bad (7)

End of Block: Perceived availability of alternatives to red meat

Start of Block: Contribution to climate change belief

Q31 How much does global red meat production (including for beef, lamb, and pork/ham) contribute to climate change?

- Not at all (1)
- A little (2)
- A moderate amount (3)
- A lot (4)
- A great deal (5)

End of Block: Contribution to climate change belief

Q33 Thinking more specifically now, please consider the list of **life events** below.   For each event, please indicate if you have experienced it at least once in **the last 2 months**:

|  | Yes (1) | No (2) | Prefer not to say (3) |
| --- | --- | --- | --- |
| Changed residential address (1) |  |  |  |
| Started a new job (2) |  |  |  |
| Retired (3) |  |  |  |
| Had a child (4) |  |  |  |
| Started co-habiting with someone (e.g., living together as a couple, with friends, etc.) (5) |  |  |  |
| Stopped co-habiting with someone (6) |  |  |  |
| Left the parental home (e.g., to start university, relocate for a job etc.) (7) |  |  |  |
| Suffered a serious illness or injury that limits your normal day-to-day activities (8) |  |  |  |
| Became unemployed (9) |  |  |  |
| Began or finished a relationship (10) |  |  |  |
| Had a significant change in friendship group (e.g., who you see or talk to, how often you see or talk to them) (11) |  |  |  |

End of Block: Life events

Start of Block: Time since life event

Display This Question:

If Thinking more specifically now, please consider the list of life events below.   For each event,... = Yes

Carry Forward Selected Choices from "Thinking more specifically now, please consider the list of life events below.   For each event, please indicate if you have experienced it at least once in the last 2 months:"

| 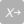 |
| --- |

Q34 Approximately how long ago was this (were these) event(s)?

|  | Less than a month ago (1) | Over a month ago, but less than two months ago (2) | Prefer not to say (3) |
| --- | --- | --- | --- |
| Changed residential address (x1) |  |  |  |
| Started a new job (x2) |  |  |  |
| Retired (x3) |  |  |  |
| Had a child (x4) |  |  |  |
| Started co-habiting with someone (e.g., living together as a couple, with friends, etc.) (x5) |  |  |  |
| Stopped co-habiting with someone (x6) |  |  |  |
| Left the parental home (e.g., to start university, relocate for a job etc.) (x7) |  |  |  |
| Suffered a serious illness or injury that limits your normal day-to-day activities (x8) |  |  |  |
| Became unemployed (x9) |  |  |  |
| Began or finished a relationship (x10) |  |  |  |
| Had a significant change in friendship group (e.g., who you see or talk to, how often you see or talk to them) (x11) |  |  |  |

End of Block: Time since life event

Start of Block: Subjective disruption from life event

Display This Question:

If Thinking more specifically now, please consider the list of life events below.   For each event,... = Yes

Carry Forward Selected Choices from "Thinking more specifically now, please consider the list of life events below.   For each event, please indicate if you have experienced it at least once in the last 2 months:"

| 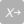 |
| --- |

Q35 For each life event, please indicate how disrupted you feel your regular, day-to-day life was during this event:

|  | Not disrupted at all | Completely disrupted |
| --- | --- | --- |

|  | 0 | 1 | 2 | 3 | 4 | 5 | 6 | 7 | 8 | 9 | 10 |
| --- | --- | --- | --- | --- | --- | --- | --- | --- | --- | --- | --- |

| Changed residential address () | 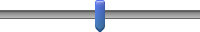 |
| --- | --- |
| Started a new job () | 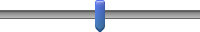 |
| Retired () | 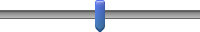 |
| Had a child () | 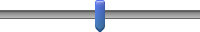 |
| Started co-habiting with someone (e.g., living together as a couple, with friends, etc.) () | 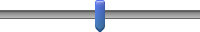 |
| Stopped co-habiting with someone () | 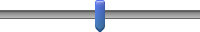 |
| Left the parental home (e.g., to start university, relocate for a job etc.) () | 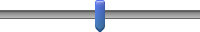 |
| Suffered a serious illness or injury that limits your normal day-to-day activities () | 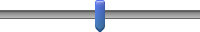 |
| Became unemployed () | 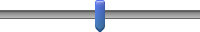 |
| Began or finished a relationship () | 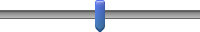 |
| Had a significant change in friendship group (e.g., who you see or talk to, how often you see or talk to them) () | 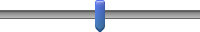 |

End of Block: Subjective disruption from life event

Start of Block: Perceived time scarcity

| 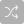 |
| --- |

Q36
Brilliant, thank you!


We’re moving to a few more general questions now before the end. Please consider the following statements and indicate the extent to which you agree or disagree with each one:

|  | Strongly agree (1) | Agree (2) | Somewhat agree (3) | Neither agree nor disagree (4) | Somewhat disagree (5) | Disagree (6) | Strongly disagree (7) |
| --- | --- | --- | --- | --- | --- | --- | --- |
| I have enough time to exercise. (1) |  |  |  |  |  |  |  |
| I have enough time to engage in hobbies or engage in ativities I enjoy. (2) |  |  |  |  |  |  |  |
| I have more to do than I have time to do it in. (3) |  |  |  |  |  |  |  |
| I have enough time to meet all of my responsibilities. (4) |  |  |  |  |  |  |  |
| Even though I am able to get done what needs to get done, I often feel like I do not have enough time. (5) |  |  |  |  |  |  |  |
| I have enough time to get done what needs to get done. (6) |  |  |  |  |  |  |  |
| I have enough time to cook healthy meals. (7) |  |  |  |  |  |  |  |
| I have enough time to spend with family/friends. (8) |  |  |  |  |  |  |  |

End of Block: Perceived time scarcity

Start of Block: Attitude towards own pro-environmental behaviour

Q37 Which of these statements best describes how you feel about your current lifestyle and the natural environment?

- I'm happy with what I do at the moment (1)
- I'd like to do a bit more to help the natural environment (2)
- I'd like to do a lot more to help the natural environment (3)

| Page Break |  |
| --- | --- |

Q38 Which of these statements best describes how you feel about your current lifestyle and the natural environment?

- I don't really do anything that is environmentally friendly (1)
- I do a few things that are environmentally friendly (2)
- I do a moderate number of things that are environmentally friendly (3)
- I do a lot of things that are environmentally friendly (4)
- I am environmentally friendly in everything I do (5)

| Page Break |  |
| --- | --- |

Q39 Please indicate the extent to which you agree or disagree with the following statements:

|  | Strongly agree (1) | Agree (2) | Somewhat agree (3) | Neither agree nor disagree (4) | Somewhat disagree (5) | Disagree (6) | Strongly disagree (7) |
| --- | --- | --- | --- | --- | --- | --- | --- |
| I think of myself as someone who is very concerned with environmental issues (1) |  |  |  |  |  |  |  |
| I would be embarrassed to be seen as having an environmentally-friendly lifestyle (2) |  |  |  |  |  |  |  |
| I would not want my family or friends to think of me as someone who is concerned about environmental issues (3) |  |  |  |  |  |  |  |
| I think of myself as an environmentally-friendly consumer (4) |  |  |  |  |  |  |  |

End of Block: Attitude towards own pro-environmental behaviour

Start of Block: Debrief and submission

Q40
**Thank you so much for all of your responses!**
 
**Your responses will be submitted and the survey will end only when you go to the next page.**
 

 That is the end of our questions! Please see below for more information about the study and who you can contact for more information.  
 
**Additional information about the study**  
Many behaviours that people undertake everyday are habitual. This means they will do them without needing to think about them. This is especially true when the person is in the place or time in which they most often perform the behaviour.
  These habitual behaviours are good for being efficient in daily activities, but they can be disrupted. Within psychology, “habit disruption” is of interest to researchers. With habit disruption, it is suggested that one way to potentially change habitual behaviours is to change (disrupt) the normal situation in which the habit is triggered. For instance, if someone uses their car to travel to work every day via the same route, but then one day their normal route is closed (changing the normal environment), they cannot rely on their habitual behaviour to get them to work. Instead, they need to think about how they are going to get to work. It is at this moment of habit disruption when new behaviours may form.
  
 We were interested to see how the extent to which people are thinking about changing their current behaviour is related to their recent experience (or lack of experience) of a life event. We expect that, compared to those whose habitual behaviour has not recently been disrupted by a life event, those whose habitual behaviour has been recently disrupted by a life event will be more likely to be thinking about a change in their behaviour.
  
 Following the analysis of the survey results, findings will be published in academic journals and presented at conferences, hopefully by the spring of 2023, although dissemination may take longer and continue for longer than this. As noted on the consent page, the information you have provided will not be identifiable in these publications or presentations. To follow this study and find out when the publications occur, please visit the project website: MOCHA (opens in new window).
  
 **Withdrawing data from the study**
 As explained on the informed consent page before the start of this survey, this survey does not ask for identifying information. As such, it will not be possible for us to identify your responses in our dataset. The inability to identify your responses means that we will be unable to withdraw your responses from the dataset.
  
 **Investigator contact details**  
 *Principal Investigator*

Email:  
                                                   
  *Co-investigator* 
  Email:

 Our postal address is:
  
 If you have any concerns related to your participation in this study, please contact the Psychology Research Ethics Committee:.
 The University of Bath is the data controller and ... is the data protection officer:.
  
 Thank you once again for contributing to our study,
 **The MOCHA research team**
 
 
**Your responses will be submitted and the survey will end only when you go to the next page.**

End of Block: Debrief and submission

**Appendix B**

Table of missing data count and percentage for all analysed variables

| **Table B1: Missing item count and percentage for investigated variables** | | | | |  |
| --- | --- | --- | --- | --- | --- |
|  | Item name | N | Missing | | |
| Variable name |  |  | Count | Percent | |
| Age (in years) | Q6_11 | 375 | 0 | .0 | |
| Personal norms 1 | Q26_1 | 372 | 3 | .8 | |
| Personal norms 2 | Q26_2 | 372 | 3 | .8 | |
| Goal intention | Q22_1 | 373 | 2 | .5 | |
| Goal feasibility | Q23_1 | 372 | 3 | .8 | |
| SRBAI 1 | Q19_1 | 373 | 2 | .5 | |
| SRBAI 2 | Q19_2 | 373 | 2 | .5 | |
| SRBAI 3 | Q19_3 | 373 | 2 | .5 | |
| SRBAI 4 | Q19_4 | 373 | 2 | .5 | |
| Health impacts | Q3 | 371 | 4 | 1.1 | |
| Self-identify | Q5 | 371 | 4 | 1.1 | |
| Formal education | Q7 | 375 | 0 | .0 | |
| Income | Q8 | 374 | 1 | .3 | |
| Changed residential address | Q33_1 | 371 | 4 | 1.1 | |
| Started a new job | Q33_2 | 371 | 4 | 1.1 | |
| Retired | Q33_3 | 371 | 4 | 1.1 | |
| Had a child | Q33_4 | 371 | 4 | 1.1 | |
| Co-habit start | Q33_5 | 371 | 4 | 1.1 | |
| Co-habit stop | Q33_6 | 371 | 4 | 1.1 | |
| Left parental home | Q33_7 | 371 | 4 | 1.1 | |
| Illness/injury | Q33_8 | 371 | 4 | 1.1 | |
| Unemployment | Q33_9 | 371 | 4 | 1.1 | |
| Relationship start/stop | Q33_10 | 371 | 4 | 1.1 | |
| Change in friendship group | Q33_11 | 371 | 4 | 1.1 | |
| Stage of change | Q21 | 373 | 2 | .5 | |
| Life event made variable | Life_event | 365 | 10 | 2.7 | |
|  |  | | | |  |

**Appendix C**

Results for regression models 1, 2, and 3 for predicting goal intention.

| Table C1. Estimates from models predicting goal intention for reducing red meat consumption with life events and antagonistic habits as moderators. | | | | | |  |
| --- | --- | --- | --- | --- | --- | --- |
| **Model 1** | **Coefficient**  **(B)** | **Standard error** | | **Lower 95% confidence interval** | **Upper 95% confidence interval** |  |
| Intercept | .59 | .60 | | -.64 | 1.72 |  |
| Health | .08 | .25 | | -.37 | .61 |  |
| Gender | -.22 | .13 | | -.48 | .04 |  |
| Age | <.01 | <.01 | | -.01 | .01 |  |
| Education | .07 | .13 | | -.19 | .33 |  |
| Income | -.04 | .15 | | -.32 | .27 |  |
| **Personal norm for red meat reduction** | **.65** | **.04** | | **.55** | **.73** |  |
| Perceived goal feasibility | .06 | .06 | | -.05 | .17 |  |
| Antagonistic habit | .03 | .04 | | -.05 | .12 |  |
| **Life event** | **.34** | **.15** | | **.05** | **.63** |  |
| **Model 2**  **(Addition of two-interactions)** | **Coefficient**  **(B)** | **Standard error** | **Lower 95% confidence interval** | | **Upper 95% confidence interval** |  |
| Intercept | .70 | .63 | -.50 | | 1.94 |  |
| Health | .05 | .24 | -.40 | | .54 |  |
| Gender | -.26 | .13 | -.51 | | .01 |  |
| Age | <.01 | <.01 | .00 | | .01 |  |
| Education | .08 | .13 | -.18 | | .35 |  |
| Income | -.03 | .15 | -.31 | | .28 |  |
| **Personal norm for red meat reduction** | **.63** | **.06** | **.50** | | **.73** |  |
| Perceived goal feasibility | .08 | .05 | -.03 | | .19 |  |
| Antagonistic habit | .02 | .05 | -.08 | | .12 |  |
| **Life event** | **.30** | **.14** | **.02** | | **.59** |  |
| **Personal norms X Antagonistic habit** | **.08** | **.03** | **.03** | | **.14** |  |
| Personal norm X Life event | .09 | .09 | -.09 | | .25 |  |
| Life event X Antagonistic habit | .07 | .10 | -.11 | | .28 |  |
| **Model 3**  **(Addition of three-way interactions)** | **Coefficient**  **(B)** | **Standard error** | **Lower 95% confidence interval** | | **Upper 95% confidence interval** |  |
| Intercept | .71 | .63 | -.50 | | 1.98 |  |
| Health | .05 | .24 | -.40 | | .54 |  |
| Gender | -.26 | .13 | -.52 | | .01 |  |
| Age | <.01 | <.01 | <-.01 | | .01 |  |
| Education | .08 | .13 | -.18 | | .35 |  |
| Income | -.02 | .15 | -.30 | | .28 |  |
| **Personal norm for red meat reduction** | **.62** | **.06** | **.50** | | **.72** |  |
| Perceived goal feasibility | .08 | .06 | -.03 | | .18 |  |
| Antagonistic habit | .02 | .05 | -.09 | | .12 |  |
| **Life event** | **.31** | **.14** | **.03** | | **.59** |  |
| **Personal norms X Antagonistic habit** | **.07** | **.03** | **.02** | | **.13** |  |
| Personal norms X Life event | .10 | .08 | -.07 | | .25 |  |
| Life event X Antagonistic habit | .08 | .10 | -.11 | | .29 |  |
| Personal norms X Life event X Antagonistic habit | .02 | .06 | -.09 | | .15 |  |
| N.B. All values in this table rounded to two decimal places. Coefficients are unstandardised.  Personal norms and antagonistic habits were mean centered for the interaction terms. | | | | | |  |
|  | | | | | | |

**Appendix D**

Results from the proportional odds and non-proportional odds forward continuation ratio models

| Table D1: Estimates and odds ratios (OR) for the **Proportional forward continuation ratio** model with logit link and Likelihood Ratio test statistics and their respective Likelihood/profile based confidence intervals (CI) | | | | | | | | |  |
| --- | --- | --- | --- | --- | --- | --- | --- | --- | --- |
|  | **Estimate** | ***p*** | **Lower 95% CI** | **Upper 95% CI** |  | **OR** | **Lower 95% CI for OR** | **Upper 95% CI for OR** | |
| Intercept 1 | **-** | **-** | **-7.58** | **-4.40** |  | **<.01** | **<.001** | **.01** | |
| Intercept 2 | **-** | **-** | **-8.59** | **-4.98** |  | **<.01** | **<.001** | **.01** | |
| **Personal norms** | **.22** | **.03** | **.02** | **.42** |  | **1.24** | **1.02** | **1.52** | |
| **Goal intention** | **1.02** | **<.001** | **.77** | **1.29** |  | **2.77** | **2.17** | **3.62** | |
| **Goal feasibility** | **.27** | **.02** | **.05** | **.49** |  | 1.30 | 1.05 | 1.63 | |
| Behavioural automaticity | -.17 | .05 | -.34 | <.01 |  | .85 | .71 | 1.00 | |
| **Life events** | **-.69** | **.02** | **-1.28** | **-.13** |  | **.50** | **.28** | **.88** | |

| Table D2: Estimates and odds ratios (OR) for the **Non-proportional forward continuation ratio** model with logit link and Likelihood Ratio test statistics and their respective Likelihood/profile based confidence intervals (CI) | | | | | | | | |
| --- | --- | --- | --- | --- | --- | --- | --- | --- |
|  | **Estimate** | ***p*** | **Lower 95% CI** | **Upper 95% CI** |  | **OR** | **Lower 95% CI for OR** | **Upper 95% CI for OR** |
| Intercept 1 | - | - | -8.74 | -4.77 |  | <.01 | <.001 | .01 |
| Intercept 2 | - | - | -8.99 | -1.22 |  | <.01 | <.001 | .30 |
| **Personal norms 1** | **.33** | **.01** | **.08** | **.59** |  | **1.39** | **1.08** | **1.80** |
| Personal norms 2 | .14 | .51 | -.28 | .56 |  | 1.15 | .76 | 1.76 |
| **Goal intention 1** | **1.11** | **<.001** | **.82** | **1.44** |  | **3.04** | **2.28** | **4.22** |
| **Goal intention 2** | **.63** | **.01** | **.12** | **1.25** |  | **1.88** | **1.12** | **3.50** |
| Goal feasibility 1 | .07 | .64 | -.21 | .34 |  | 1.07 | .81 | 1.41 |
| **Goal feasibility 2** | **.75** | **<.001** | **.32** | **1.24** |  | **2.11** | **1.37** | **3.44** |
| Behavioural automaticity 1 | .03 | .76 | -.18 | .26 |  | 1.04 | .83 | 1.29 |
| **Behavioural automaticity 2** | **-.59** | **<.001** | **-.96** | **-.28** |  | **.55** | **.38** | **.76** |
| **Life events 1** | **-.99** | **<.01** | **-1.74** | **-.29** |  | **.37** | **.17** | **.75** |
| Life events 2 | -.16 | .78 | -1.31 | .95 |  | .85 | .27 | 2.60 |
| Note: 1 = Predictors’ partial estimates for the Predecisional vs Pre/actional category membership; 2 = Predictors’ partial estimates for the Pre/actional vs Postactional membership. | | | | | | | | |

| Table D3. Fit statistics and likelihood ratio tests for the Proportional, non-proportional, and partial proportional forward continuation ratio models. | | | |
| --- | --- | --- | --- |
| **Model** | **AIC** | **BIC** | **Likelihood ratio test against proportional odds model** |
| Proportional odds | 366.89 | 394.00 | - |
| Non-proportional odds | 349.89 | 396.35 | χ²(5) = 27.00, *p* < .001 |
| Proportional, with non-parallel personal norm | 366.16 | 397.13 | χ²(1) = 2.74, *p* = .10 |
| Proportional, with non-parallel goal intention | 362.42 | 393.40 | χ²(1) = 6.47, *p* = .01 |
| Proportional, with non-parallel goal feasibility | 358.38 | 389.35 | χ²(1) = 10.51, *p* < .01 |
| Proportional, with non-parallel behavioural automaticity | 355.09 | 386.07 | χ²(1) = 13.80, *p* < .001 |
| Proportional, with non-parallel life event | 368.80 | 399.77 | χ²(1) = .10, *p* = .76 |
| Partial proportional model | 347.85 | 386.57 | χ²(3) = 25.04, *p* < .001 |
| **Model** | **AIC** | **BIC** | **Likelihood ratio test against *non-*proportional odds model** |
| Partial proportional model | 347.85 | 386.57 | χ²(2) = 1.96, *p* = .38 |
| **Model** | **AIC** | **BIC** | **Likelihood ratio test against the partial proportional model** |
| Null (intercept only) model | 547.26 | 555.00 | χ²(8) = 215.41, *p* < .001 |
